# Practical Cluster Models for a Layered β-NiOOH Material

**DOI:** 10.3390/ma10050480

**Published:** 2017-04-29

**Authors:** Valeria Butera, Maytal Caspary Toroker

**Affiliations:** Department of Materials Science and Engineering, Technion—Israel Institute of Technology, Haifa 3200003, Israel; valeria.butera@unical.it

**Keywords:** nickel oxides, density functional theory (DFT), water splitting, cluster models, ECPs

## Abstract

Due to the high oxygen evolution reaction (OER) activity, stability, and abundance of NiOx materials, they are found to be promising catalysts, competitive with expensive metal oxides such as IrO_2_ and RuO_2_. From a theoretical point of view, studies reported in the literature so far are mostly based on density functional theory using periodic slab models for the bulk and surface of β-NiOOH, one of the active NiOx phases. However, cluster models are a valid method to investigate many aspects about structure, charge carrier transport properties, and OER activity of β-NiOOH. Hence, here we present new cluster models for the surface of β-NiOOH, where the oxygen atoms are bonded to Mg effective core potentials (ECPs) mimicking neighboring atom cores. This cluster embedding procedure is superior to saturating the cluster with hydrogen atoms, and to using other atomic ECPs for β-NiOOH. We find that layered materials such as β-NiOOH are more vulnerable to geometrical rupture and therefore a cluster approach requires additional care in choosing the embedding approach. We evaluated the models by using them to calculate the energy required for water adsorption and deprotonation, which are essential ingredients for OER. Specifically, our results agree with previous slab models that the first deprotonation reaction step requires a large amount of energy. In addition, we find that water and hydroxyl groups have high adsorption energy and therefore the first deprotonation step is limiting the reaction efficiency.

## 1. Introduction

Because of the constantly growing demand for energy, along with increasing pollution, the need for renewable energy sources as alternatives to fossil-fuel-based technologies is significant [1,2,3,4,5]. One promising way is to use a photo-electrochemical cell to split water into H_2_ fuel and O_2_ gas during the hydrogen evolution reaction (HER), at the cathode and the oxygen evolution reaction (OER), and at the anode, respectively [6,7,8]. The latter is a far more complex process that proceeds through four-proton-coupled electron transfer reactions, and is energetically inefficient [9,10,11]. Identifying active catalysts that accelerate the reaction and reduce the overpotential remains a key challenge. In this context, NiOx materials have been proven to be competitive catalysts for precious metal oxides such as IrO_2_ and RuO_2_ thanks to their high OER activity, stability, and abundance [7,12,13,14,15].

X-ray diffraction experiments have shown that NiOx consists of four different phases: β-Ni(OH)_2_, α-Ni(OH)_2_, β-NiOOH, and γ-NiOOH [16,17]. The most important similarity between α-Ni(OH)_2_ and γ-NiOOH is the presence of intercalated species [18,19,20,21,22,23], which make these phases more difficult to investigate with respect to both of the β-phases. In addition, β-NiOOH is generally reported as the active OER phase [24,25], even though recently Bediako et al. [26] suggested that γ-NiOOH may be more efficient. Selloni and coworkers [27] have identified the stable structures of β-NiOOH using a genetic algorithm approach combined with dispersion-corrected hybrid density functional theory calculations. The authors found that the most favorable structures consisted of alternating layers of NiO_2_ and β-Ni(OH)_2_.

One of the most interesting findings is that the OER overpotential can be significantly lowered by Fe impurities [28,29]. This discovery pushed many researchers to investigate the phenomenon, optimizing Fe content and synthesizing various Ni-Fe mixed compounds [30,31,32,33]. In this context, various computational studies attest that Fe is the active site in either one of NiOOH’s phases: β-NiOOH or γ-NiOOH phases [29,34,35]. Thanks to both theoretical and experimental investigations of pure and Fe-doped β-NiOOH, several aspects about the OER activity have been understood [29,34,35]. However, the theoretical studies are all based on density functional theory using periodic slab models for the NiOOH bulk and surface [34,35]. To the best of our knowledge, from a theoretical point of view, no study has used density functional theory (DFT) with a cluster approach to study β-NiOOH surface. However, cluster approach investigation has several advantages over a periodic slab model, including the ability to alter the total charge without interaction with periodic images, the compatibility with the existing molecular orbital methods, and computational efficiency.

Therefore, cluster approach calculations are a valid and promising method that can be used to investigate the OER reaction mechanism on pure and Fe-doped β-NiOOH. In a previous work [36], in fact, we have used similar cluster model sizes to simulate the bulk structure of pure and Fe-doped β-Ni(OH)_2_ in order to investigate some electronic properties such as the band gap, the chemical nature of highest occupied molecular orbital/lowest unoccupied molecular orbital (HOMO/LUMO), and the charge density difference as a result of excess charge carriers. Using a cluster approach model, our studies contributed to shed light on the potential role of the Fe dopant in β-Ni(OH)_2_.

On the basis of these important results, we decided to use, for the first time, a cluster model approach to investigate the surface of β-NiOOH. However, to perform such calculations, reasonable cluster models first need to be constructed. Different procedures can be used in order to replace the excess positive charges of the Ni cations immediately next to the O anions at the edge of the clusters. Examples for procedures include terminating the cluster with H atoms [37] and capping the cluster with electron core potentials, ECPs [38]. Our investigation shows that the only method that leads to stable structures, especially when a water molecule and OH group are added, consists of capping the cluster with Mg ECPs. Following this procedure, we built clusters of three different sizes in which three, four and five Ni atoms are present, respectively, and with linear and non-linear conformations for each. The clusters are evaluated by calculating Ni-O bond lengths, water and OH adsorption, and water deprotonation, which is the first reaction step for OER.

## 2. Methods and Computational Protocol

All the calculations were performed using the Gaussian 09 quantum chemistry package [37,39]. The default “Berny algorithm” developed by Bernhard Schlegel was used for all the atomic structure optimizations [38,40]. The more reliable quadratically convergent SCF procedure was used.

According to previous nickel oxide cluster calculations [41], LANL2DZ [38,42,43,44] pseudo potential for Ni and Fe atoms and the fully electron basis sets 6-311++G** for O and H were chosen. All the cluster geometries were optimized with the PBE0 [45,46,47] functional that contains 25% Hartree-Fock (HF) exchange and the Perdew-Burke-Ernzerhof (PBE) exchange-correlation functional. In a previous work [27], the PBE0-DF3 functional was used to correct the underestimated band gap and to account for Van der Waals effects expected in the bulk structures investigated by the authors. We note that our cluster models consist of just one layer, since in our previous publication [36] we have already shown that bulk single-layered clusters of β-Ni(OH)_2_ have converged electronic properties relative to the number of layers. Hence, Van der Waals interactions are not important in our investigation and the choice of PBE0 is reasonable.

## 3. Results and Discussion

Our main results comprise the construction of new cluster models for the β-NiOOH surface. The central novelty is that in a layered material there is a problem of cluster stability and it is not at all obvious what the solution is (for example, which ECP to use). We present a thorough analysis of several attempts to solve this problem (as detailed extensively in the supporting information), in addition to comparing with periodic slab models.

We built clusters consisting of Ni^3+^, O^2−^ and H^+^ ions, carved out from the optimized periodic structure of β-NiOOH surface reported by Selloni and co-workers [28], whose optimized structure shows almost identical lattice parameters to the experimental structure [48,49,50]. We followed several procedures for embedding the cluster, as detailed in the supporting information, and found that the structure of the clusters collapse during geometry optimization when hydrogen atoms or non-Mg atomic ECPs are used. Our calculations show that in order to obtain stable optimized cluster geometries Mg ECPs can be used to replace Ni^+3^ cations at the edge of the cluster and they need to be kept fixed during the optimization.

**Figure 1:**
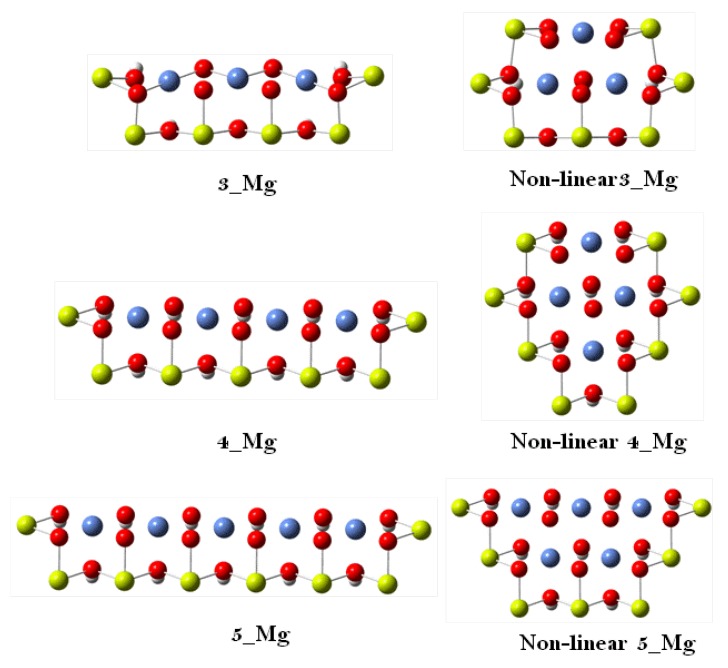
Cluster models for β-NiOOH consist of *X* = 3, 4 and 5 Ni cations, denoted by *X*_Mg and Non_linear *X*_Mg for the linear and non-linear structures, respectively. GaussView software (version 5.0.8, Semichem Inc., Shawnee Mission, KS, USA) [55] was used for visualization of the cluster structures. Red, blue, white, and green spheres represent O atoms, Ni atoms, H atoms, and Mg ECPs, respectively.

The positive point charges of the outer Ni^+3^ ions were substituted by effective core potentials [51]. The choice of the ECPs usually depends on the radii and the cations oxidation states. Charging/discharging experiments performed by Bamard et al. [52] showed that the Ni oxidation state in β-NiOOH is 2.7–3.0 while X-ray photoelectron spectroscopy (XPS) revealed the Ni^+2^ oxidation state [53,54]. The interpretation of oxidation states from these various experiments has been mentioned in reference [28] and references therein, but theoretical investigations of β-NiOOH suggested that not all Ni atoms in the crystal structure have a +3 oxidation state [27]. Ni^+2^ and Ni^+3^ oxidation states were also found, suggesting a disproportion of half of the Ni^+3^ cations to Ni^+2^/Ni^+4^ pairs. Our own recent theoretical investigations [35] using Bader charge analysis and periodic DFT confirm a dominance of Ni^+3^ oxidation states and some Ni^+2^ and Ni^+4^ cations. In this study, a variance in oxidation states for Ni is confirmed through Mülliken spin analysis (in the smallest clusters, without adsorbed molecules, the spin corresponds to oxidation states +3 and +4 of Ni; and for the largest cluster there is also a +2 oxidation state of Ni). The choice of Mg ECPs is indeed a non-trivial approach for embedding this material, since the Ni atoms contain several oxidation states; among them Ni^+2^ appear in smaller amounts than Ni^+3^. Mg ECPs would be the natural choice for replacing Ni^+2^ cations around the cluster, assuming Mg^+2^ is mimicked by the Mg ECPs. Other choices for replacing Ni ions include Al and Si ECPs, representing Ni^+3^ and Ni^+4^, respectively, but these options were shown to fail at maintaining the stability of the cluster during geometry optimization (see supporting information); hence, our final choice of Mg ECPs is just practical. Following this procedure of adding Mg ECPs, we built clusters of three different sizes, as reported in Figure 1. Those clusters consist of three, four and five Ni atoms, respectively. For each size, linear and non-linear conformations have been investigated.

In Table 1, we report the averaged computed Ni–O distances of the Ni–OH and Ni–O bond lengths for all the cluster sizes. In the same table, the experimental values are given. In particular, non-linear cluster models give very similar bond distances values for the Ni–OH for all the considered cluster models. However, these cluster models tend to overestimate the Ni–O bond distances more than the linear cluster model. Nevertheless, the bond distances of the periodic cluster models (obtained from initial geometries of the clusters cleaved from periodic slabs) are 1.990 and 1.981 Angstrom for the Ni–OH and Ni–O distances, respectively; hence, our cluster models agree better than the slab models with the experimental results. Increasing the cluster model size does not affect the trend and no significant differences are observed showing that even the smallest cluster can be used for the catalyst modeling. This important aspect is also observed when the deprotonation energy is taken into account as we will discuss later. The bond distances are a necessary, but not a sufficient, condition for these clusters to be appropriate for modeling OER. Hence, we perform surface adsorption energy calculations of the most influential reaction intermediates of OER (involving the reaction step requiring the largest energy [28]).

**Figure 2:**
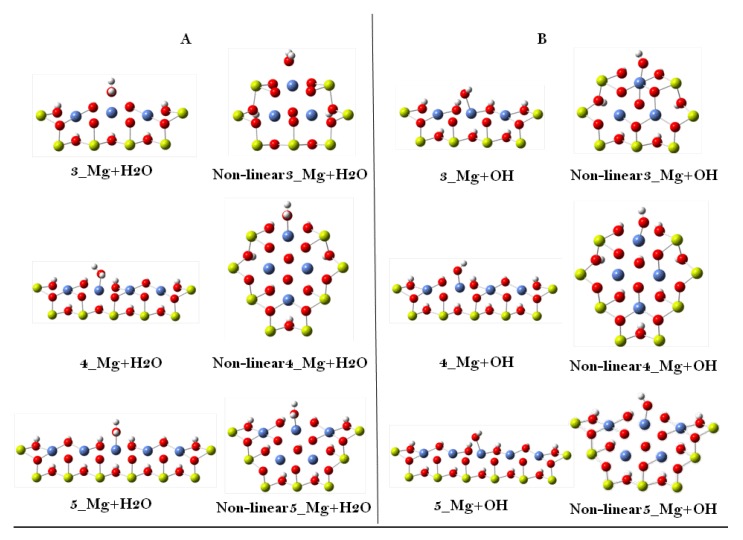
Cluster model of 3_Mg, 4_Mg, 5_Mg and non-linear 3_Mg, 4_Mg, 5_Mg with coordinated H_2_O (**A**) and OH (**B**). GaussView software, (version 5.0.8, Semichem Inc., Shawnee Mission, KS, USA) [55] was used for visualization of the cluster structures. Red, blue, white, and green spheres represent O atoms, Ni atoms, H atoms, and Mg ECPs, respectively.

In addition, our calculations show that when a water molecule is added to the system it coordinates to the Ni metal (see Figure 2A). A similar result is found when the water molecule is replaced by an OH group (see Figure 2B).

With these clusters, the adsorption energies, E_ads_, of the water molecule and of the OH group on the Ni cation are computed using the following equation:

E_ads_ = (E_cluster_Mg_ + E_H_2_O/OH_) − E_cluster_Mg + (H_2_O/OH)_

where E_cluster_Mg_ is the energy of the optimized cluster, E_H_2_O/OH_ is the energy of the water molecule or a neutral OH group, and E_cluster_Mg +(H_2_O/OH)_ is the energy of the cluster when the water molecule or the OH group is coordinated to the Ni center. As can be seen from Table 2, in which all the calculated values for all the three different cluster sizes and for both the linear/non-linear conformations are reported, both the water molecule and OH group are strongly coordinated to Ni. This implies that deprotonation would be difficult due to the strong bonding of reaction intermediates. In fact, the binding energies of H_2_O and of OH are directly proportional to the energy required for deprotonation.

**Scheme 1:**
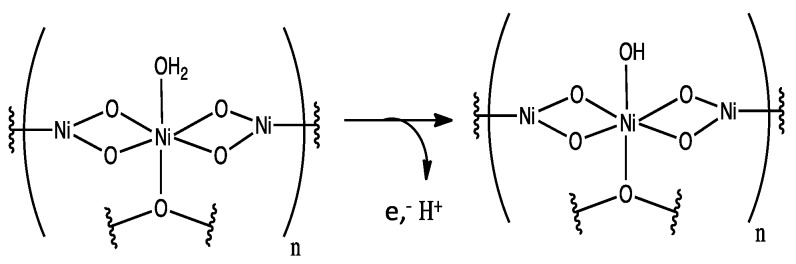
First reaction deprotonation step of the OER mechanism for β-NiOOH.

Another calculation that we performed with these clusters was the energy required for the first deprotonation step. The first step of the OER mechanism with β-NiOOH, as proposed by Selloni et al. [28], is the release of a proton and an electron from an adsorbed water molecule, leaving an adsorbed OH (see Scheme 1).

We compute the deprotonation energy according to the reaction:

Cluster_Mg+H_2_O→Cluster_Mg+OH+1/2 H_2_

as follows:

E_dep_ = (E_cluster_Mg+OH_ + 1/2 E_H2_) − E_cluster_Mg+(H_2_O)_

where E_cluster_Mg+OH_ is the total energy of a cluster with adsorbed OH, E_H2_ is the total energy of the isolated H_2_ molecule, and E_cluster_Mg+(H_2_O)_ is the total energy of a cluster with adsorbed H_2_O. Table 3 reports all the calculated deprotonation energies. Our results show that when increasing the size of the cluster, the computed deprotonation energies do not change significantly when both the linear and non-linear cluster models are used. In addition, for equal numbers of Ni atoms, non-linear clusters give larger energy values in comparison to linear ones for all three different sizes. These results lead us to conclude that the presence of the additional atoms has no relevant importance in the stabilization of the coordinated water molecule, or on the deprotonation OH product. Therefore, as already anticipated, the consistency of the deprotonation energy with cluster size shows that even small clusters can be used for catalysis modeling. The changes in Mülliken spin analysis also shows reasonable oxidation states for the Ni atom that binds to the adsorbates: +3 for the cluster without adsorbates or with H_2_O and +2 after deprotonation.

Our calculated deprotonation energies using cluster models agree with previous periodic slab calculations. Selloni et al. [28] have computed an energy of 2.06 eV (∆G = 1.69 eV) using Spin-polarized density functional theory (DFT) in the plane wave basis set and ultrasoft pseudopotetial framework, as implemented in QUANTUM-ESPRESSO software. Furthermore, the first deprotonation step was found to have the highest energy difference, and limits the reaction efficiency. We also obtain substantial values for the first deprotonation step.

## 4. Conclusions

Building clusters for a solid that includes stacked layers is especially challenging. The crystal structure of β-NiOOH has layers that are weakly bound with inter-layer voids. Such a structure produces clusters that are unstable and tend to strongly reconstruct and geometrically collapse. However, a cluster approach treatment has several advantages over a periodic slab model, including the ability to alter the total charge without interacting with periodic images, the compatibility with the existing molecular orbital methods, and computational efficiency.

We explored several procedures in order to construct reasonable clusters for β-NiOOH. According to the first procedure, the oxygen anions at the edge of the clusters are saturated by adding protons in a way that preserves the Ni–O bond vectors of the crystal. In the second procedure, the oxygen atoms are bonded to selected ECPs mimicking atom cores. Our calculations show that stable optimized cluster model geometries can be obtained only when Mg ECPs are used. We note that the thermal stability of similar cluster models has been tested for other materials using Molecular Dynamics [56], but this technique is beyond the scope of this study.

Afterwards, following the selected procedure, we built clusters with different sizes. The comparison between the experimental Ni–O bond lengths and our computed values shows that increasing the cluster size has no significant influence on the bond distances. In addition, the water deprotonation energies of each cluster model is compared to that obtained by previous slab calculations. In agreement with previous calculations, we find that the deprotonation energy is high (above 2 eV). Also, in this case, we observed that, when increasing the size of the cluster, the computed deprotonation energies were very similar when both the linear and non-linear cluster models were used. Hence, we constructed reasonable cluster models that are geometrically stable and adequate for catalysis modeling.

The adsorption energies of the water molecule and the OH group were also calculated showing that both the water molecule and the OH group are strongly coordinated to the Ni center. Hence, we conclude that the relative adsorption energy of reaction species limits the efficiency of the first deprotonation reaction step.

We anticipate that these clusters can be further used for subsequent analysis of OER. Specifically, the main advantage of cluster models over periodic slab models is the ability to add excess electron or hole charge without errors arising from periodic images, as we demonstrated in our recent work on clusters for the bulk [36]. Moreover, the small size of the clusters will allow using more computational demanding correlated methods beyond DFT [57]. This study provides the basis for future work on modeling OER for layered materials.

## Figures and Tables

**Table 1:** Ni–O distance of the Ni–OH and Ni–O bond lengths for all the three cluster sizes in their linear and non-linear structures alongside the experimental bond lengths. Units in Angstrom.

	3		4		5		Slab Model	Experimental
	Linear	Non-Linear	Linear	Non-Linear	Linear	Non-Linear		
Ni–OH	2.243	2.075	2.191	2.038	2.228	2.066	1.990	2.060
Ni–O	1.912	1.947	1.909	1.961	1.914	1.938	1.981	1.875

**Table 2:** Adsorption energy values of water molecule on Ni atom in eV for β-NiOOH cluster models capped with Mg ECPs.

Cluster Model		Adsorption Energy (eV)		
		Cluster 3_Mg	Cluster 4_Mg	Cluster 5_Mg
Linear	H_2_O	1.868	1.264	1.406
	OH	1.412	1.455	1.441
Non-linear	H_2_O	1.744	1.545	1.555
	OH	1.818	1.130	1.177

**Table 3:** Deprotonation energy values in eV for all the studied β-NiOOH cluster models capped with ECPs.

Cluster Model	Deprotonation Energy (eV)		
	Cluster 3	Cluster 4	Cluster 5
Linear	2.889	2.772	2.656
Non-linear	3.419	3.379	3.341

## Supplementary Materials

The following are available online at http://www.mdpi.com/1996-1944/10/5/480/s1, Figure S1: Cluster models used for β-NiOOH in which (**a**) the oxygen atoms on the edge are saturated by hydrogen atoms: 1_H and 3_H, containing 1 Ni atom and 3 Ni atoms, respectively; (**b**) the oxygen atoms on the edge are saturated by Al ECPs: 1_Al, 3_Al, Non-linear3_Al, 4_Al, 6_Al and 12_Al containing 1, 3, 4, 6, 12 Ni atoms, respectively. Figure S2: Partially relaxed geometry of 3_H cluster model. Figure S3: Partially relaxed geometry of 1_Al cluster model. Figure S4: Three layers of 1_Al cluster model: (**a**) starting geometry showing the active site; (**b**) partially relaxed geometry. Figure S5: Final optimized geometry of non-linear 3_Al cluster model. Figure S6: Partially relaxed geometry of the three layers non-linear 3_Al cluster model. Figure S7: Partially relaxed geometry of the three layers non-linear 3_Al cluster model using ECPs to describe all the atoms of the model except the active site. Figure S8: Bent partially relaxed geometry of cluster model 12_Al. Figure S9: Mixed ECPs cluster models: (**a**) starting geometry and (**b**) final geometry for cluster 3_Al_2__Si_Mg_3_; (**c**) starting geometry and (**d**) final geometry for cluster model 3_Al_Mg_4_.
