# Supplementary material for: Practical Cluster Models for a Layered β-NiOOH Material
Source: Materials (Basel). 2017 Apr 29;10(5):480. doi: 10.3390/ma10050480 (PMC5459011; doi:10.3390/ma10050480)
Supplement: Supplementary file 1 [file materials-10-00480-s001.pdf]

Supporting Information: Practical Cluster Models for a Layered β-NiOOH Material

Valeria Butera and Maytal Caspary Toroker

Cluster Description

SI-1. First Procedure
- Results and Discussion
- Cartesian Coordinates (Å) of Initial, Partially Relaxed and Final Optimized Geometries of Cluster Model a)

SI-2. Second Procedure
- Results and Discussion
- Cartesian Coordinates (Å) of Initial, Partially Relaxed and Final Optimized Geometries of Cluster Model b)
- Cartesian Coordinates (Å) of Initial, Partially Relaxed and Final Optimized Geometries of Mixed ECPs Clusters

SI-3. Cartesian Coordinates (Å) of Optimized Geometries of Mg ECPs Cluster Models
Si-4. Example of Input File
SI-5. References

Cluster Description

All the cluster structures that have been studied in this work are reported in Figure S1. Two different procedures were investigated in order to replace positive charges of the Ni cations immediately next to the O anions of the clusters. According to *the first procedure* [1], protons were added along the Ni–O bond vectors, replacing Ni atoms originally present in the crystal structure in order to preserve the symmetry of β-NiOOH. Two different cluster sizes have been investigated following the *first procedure* and reported in Figure S1, cluster model a). All the details about the first procedure are discussed in the first section of the SI (SI-1).

In a *second procedure* [2] the positive point charges of the outer Ni cations were substituted by effective core potentials (ECPs), including Al, Mg and Si ECPs Detailed information about cluster models containing only Al- and mixed Al/Mg/Si-ECPs will be discussed in the second section of the SI, SI-2. Cluster model b), reported in Figure S1, refers to clusters containing Al ECPs. For all the clusters, we adopt labels in which the *number* indicates the number of Ni atoms, *H* indicates the cluster saturated by adding protons, while *Al* represents that the cluster is saturated by Al ECPs.

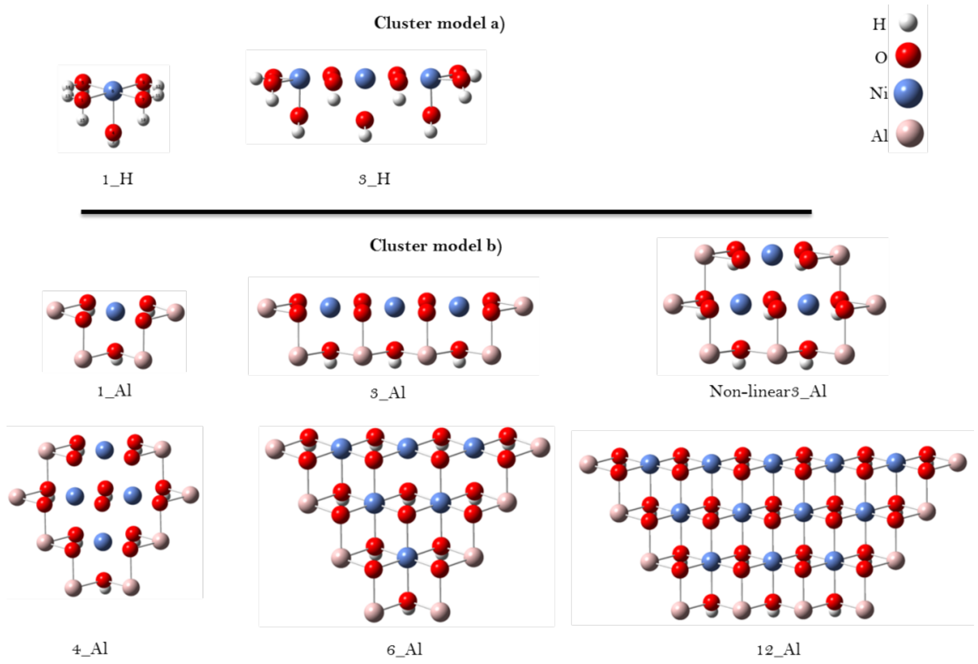

Figure S1. Cluster models used for β-NiOOH in which (**a**) the oxygen atoms on the edge are saturated by hydrogen atoms: 1_H and 3_H, containing 1 Ni atom and 3 Ni atoms, respectively; (**b**) the oxygen atoms on the edge are saturated by Al ECPs: 1_Al, 3_Al, Non-linear3_Al, 4_Al, 6_Al and 12_Al containing 1, 3, 4, 6, 12 Ni atoms, respectively. GaussView software [3] was used for visualization of the cluster structures.

SI-1. First Procedure

Results and Discussion

The smallest cluster that can be carved out of the crystal structure of the β-NiOOH surface while having correct chemical coordination contains just one Ni atom bonded to five O^{2-} anions. According to the first procedure [1], the charged O atoms were saturated by adding protons in a way that preserved the crystal structure and yielded the final composition NiH_9O_5 (see Figure S1, cluster 1_H). Only bond lengths were optimized, while keeping fixed the dihedral angles that include the added protons in order to maintain the directionality of all the bonds in the cluster to that of the β-NiOOH structure.

As we can see from cluster 1_H in Figure S1, after the addition of the protons, the resulting cluster includes four water molecule coordinates to the Ni atom in the equatorial position and one OH group coordinated in the axial position far away from the active site. After performing the cluster optimization, the equatorial Ni-O bond distances changed, becoming about 0.02 Å shorter with respect to the starting geometry.

The relaxation of the cluster was also performed while adding a hole to the system and maintaining the fixed dihedral angles. Detecting the location of the excess hole in the cluster is important, since this is the charge carrier that is involved in the OER. In this case the equatorial Ni-O bond distances were found to be about 0.2 Å longer with respect to the neutral cluster. The distance between the Ni atom and the axial O atom that is connected to a single H atom is, instead, just 0.04 longer. From the spin density analysis, we found that the hole is located on the axial O atom that has a hydrogen atom absent with respect to the other equatorial O atoms. The absence of H atoms leads to the occupation of the hole. Hence, the location of the hole is incorrectly affected by the presence of the OH group on the opposite side away from the active site.

We performed the calculation, adding a water molecule to the system. Our calculation shows that a water molecule is coordinated to the available site above the Ni atom with a bond distance of 2.09 Å. This distance does not change when a hole is added to the cluster. As we obtained previously,

the hole is again located on the axial O atom belonging to the OH group. This occurs also when the added water molecule is substituted for an OH group. Therefore, all our calculations involving such a cluster that show that the hole is located on the oxygen of the OH group in axial position cannot correctly describe the surface.

We saturated the oxygen atom in axial position by adding an extra proton, yielding the final composition of $NiH_{10}O_5$ in order to verify if the addition of H on this atom could lead to a different location of the hole in the cluster. However, when we added the second water molecule that should be coordinated to the Ni in an available axial position, that molecule went away from the cluster after geometry optimization.

The calculations presented so far show that this simple cluster model is not suitable for investigating β-NiOOH material. For this reason, we decided to move to a bigger cluster model, which contains 3 Ni atoms. However, the optimization of the geometry maintaining the dihedral angles fixed in order to preserve the directionality of the Ni-O bond vectors of the original cluster, ended with non-convergence error. The last geometry of the optimization cycles (before the calculation is interrupted with this error) is reported in Figure S2. As we can see from Figure S2 the final geometry is completely distorted and bent with respect to the original one.

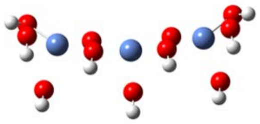

Figure S2. Partially relaxed geometry of 3_H cluster model. GaussView software [3] was used for visualization of the cluster structures.

Selloni and co-workers [4] have performed density functional theory calculations in the plane wave and ultrasoft pseudopotential frameworks as implemented in QUANTUM-ESPRESSO on several surfaces of pure β-NiOOH and Fe-doped β-NiOOH in order to map out the OER reaction pathway. In their calculations, they considered a monolayer of water molecules adsorbed on the Ni atoms. On the basis of their work, we tried to optimize our cluster *in presence of just one coordinated water molecule and also a monolayer* as they suggest. The water molecules, in fact, should occupy the available sites of the exposed Ni atoms and establish hydrogen bonds with the oxygen of the cluster. It is expected then that both of these aspects could enhance the stability of the cluster. However, our expectations were disappointed and the calculations ended without reaching the geometrical convergence and maintaining the correct crystal structure.

Hence, even the bigger cluster with three Ni atoms is not suitable as cluster model for the investigation of NiOOH surface by a cluster approach when the first procedure of adding hydrogen atoms is followed. For this reason, we decided to investigate the second procedure that will be described in the next section.

Cartesian Coordinates (Å) of Initial, Partially Relaxed and Final Optimized Geometries of Cluster Model a)

Initial geometry of cluster model 1_H

O	1.49450000	-1.24348400	-0.42424400
H	0.00000000	0.16341600	2.42482200
H	1.49450000	2.08596000	0.69149700
O	0.00000000	-0.56995000	1.61139800
O	1.49450000	1.35259400	-0.12192700
Ni	0.00000000	0.04871600	-0.27956200
O	-1.49450000	-1.24348400	-0.42424400
H	-1.49450000	2.08596000	0.69149700
O	-1.49450000	1.35259400	-0.12192700

H	-1.49449900	-1.54895500	0.48585900
H	-2.21875100	-0.61727000	-0.35413000
H	-2.21561300	0.72345800	-0.19798800
H	2.21875100	-0.61727000	-0.35413000
H	2.21561300	0.72345900	-0.19798800
H	1.49450000	-1.54895600	0.48585800

Partially relaxed geometry of cluster model 1_H

O	-1.36472900	1.32619400	-0.45363600
H	0.00001100	-0.08075100	2.35929300
H	-1.50248800	-2.02457700	0.64837500
O	-0.00002700	0.56026800	1.62673800
O	-1.48325800	-1.35981400	-0.06088900
Ni	0.00000200	-0.07788600	-0.23013800
O	1.36458800	1.32632400	-0.45362700
H	1.50270900	-2.02442200	0.64836200
O	1.48340200	-1.35966100	-0.06090200
H	1.29667700	1.96017500	0.28533000
H	2.28486400	1.00587200	-0.42459500
H	1.85262100	-1.78374600	-0.85755100
H	-2.28497600	1.00565600	-0.42460000
H	-1.85242300	-1.78394600	-0.85753800
H	-1.29687600	1.96005200	0.28532100

Initial geometry of cluster model 3_H

O	1.48539900	-1.18217800	-0.54929000
H	-0.03474700	-0.15344900	2.30670100
H	1.47583900	2.01597400	0.90047200
O	-0.02815800	-0.71805300	1.53031300
O	1.48333600	1.36947500	0.01646500
Ni	-0.01000600	0.09041400	-0.28752400
O	-1.50346700	-1.17835200	-0.57743600
H	-1.51302500	2.01979900	0.87232600
O	-1.50552800	1.37330000	-0.01168100
Ni	-2.99887100	0.09423900	-0.31567100
Ni	2.97886100	0.08658800	-0.25937800
O	4.26797600	-1.13857700	-0.59590700
H	5.02641700	-0.57421800	-0.42897600
O	4.34174700	1.26116400	-0.06186700
O	-4.15328300	-1.28502400	-0.51831800
H	-4.95283800	-0.76141700	-0.42805800
O	-4.28526100	1.32613700	0.00639800
H	-4.32017900	1.79511400	0.84332200

O	3.02089100	-0.77483400	1.33193700
H	2.96144100	-0.27556600	2.14973700
O	-3.02341200	-0.70082800	1.31017400
H	-3.09313600	-0.16659600	2.10473900
H	4.48876700	1.68775800	0.78548300

Partially relaxed geometry of cluster model 3_H

O	1.69910900	-0.69653000	-1.10638100
H	-0.02966000	-2.21638900	1.66575300
H	1.64954100	0.88515800	1.79288400
O	-0.00677300	-2.02849200	0.72410800
O	1.55494300	0.84578100	0.85990500
Ni	-0.00235800	-0.19106700	-0.02543700
O	-1.66348600	-0.73099100	-1.08883200
H	-1.67533100	0.91339900	1.76824700
O	-1.53753700	0.87148400	0.83989000
Ni	-3.07909300	0.23392900	-0.16349200
Ni	3.08731700	0.24833400	-0.15363700
O	4.33727100	0.04897500	-1.54150900
H	5.00408700	0.60696100	-1.08245400
O	4.27173900	1.60321100	0.52354400
O	-4.33443000	-0.01339500	-1.56722800
H	-4.97094800	0.61472600	-1.16018000
O	-4.29074100	1.61791800	0.45588800
H	-4.32939900	1.52122900	1.41221900
O	3.69991800	-1.39979400	0.88892200
H	3.63363700	-1.31978400	1.84971900
O	-3.73235300	-1.30470700	1.00190600
H	-3.81598600	-1.16011800	1.95239900
H	4.38851600	1.49364500	1.47155600

SI-2. Second Procedure

Results and Discussion

Following a procedure reported previously in literature [2], we replaced the positive point charges immediately next to the O^{2-} anions in the clusters by effective core potentials, ECPs. The ECPs are chosen according to previous experimental and theoretical results on the Ni oxidation states that show several possible oxidation states for Ni: +2, +3 and +4 [5-8]. Taking this into account, three different ECPs can be used. Al ECPs can replace Ni^{3+} because of the similar ionic radii of Ni^{+3} and Al^{3+}. In a similar way, Ni^{2+} and Ni^{4+} can be replaced by Mg ECPs and Si ECPs, respectively. We perform calculations in which all Ni atoms of the clusters are replaced by Al ECPs or Mg ECPs as well as a combination of Al, Mg and Si ECPs.

Al ECPs

In a first attempt, all Ni cations were replaced by Al ECPs. Also in this case, we first focused our attention on the smallest cluster, 1_Al. In our first try, we kept fixed just the Al ECPs atoms. However,

the optimization ended without reaching the geometry convergence. As we can see from Figure S3, in fact, the last obtained geometry before the calculation ends is entirely distorted from the starting geometry of the cluster. Unfortunately, the calculation performed fixing the dihedral angles alongside the Al ECPs and allowing the bond distances to change leads to similar results. Also in this case, adding a water molecule on the vacant site of the Ni atom, so that Ni can be fully coordinated does not change the final result during geometry optimization and the added water molecule leaves the Ni site and goes away.

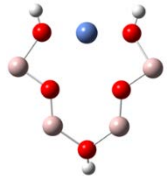

Figure S3. Partially relaxed geometry of 1_Al cluster model. GaussView software [3] was used for visualization of the cluster structures.

We tried considering three different layers with the aim of establishing hydrogen bond interaction between the layers, whichshould prevent the cluster collapsing during the optimization (see Figure S4, a). However, the calculation in which we fixed just the Al ECPs didn't reach the convergence and the cluster model collapsed after a few optimization cycles. We tried, therefore, fixing all the atoms of the model except that belonging to the active site (see selected atoms in Figure S4 a). As seen in Figure S4 b) Ni and H atoms that are not fixed move away during the optimization.

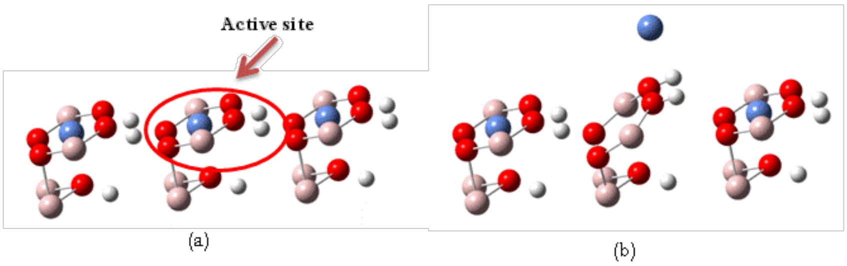

Figure S4. Three layers of 1_Al cluster model: (**a**) starting geometry showing the active site; (**b**) partially relaxed geometry. GaussView software [3] was used for visualization of the cluster structures.

The dissatisfying results obtained so far pushed us to increase the size of the cluster model with the hope this could help to reach the convergence criteria. Similar results have been obtained also when the bigger cluster with three Ni atoms linearly arranged in the same layer, 3_Al, has been investigated. Also in this case, even if all the Al ECPs atoms and all the hydrogen are kept fixed during the optimization, the calculation ends without reaching the convergence criteria.

A non-linear cluster containing three Ni atoms, non-linear 3_Al, has been investigated. Keeping fixed all the Al ECPs and H atoms of the cluster model, we got the final optimized structure (Figure S5). Adding a water molecule to the Ni center leads to the same results we got for the previous clusters. In fact, during the optimization calculation H_2O moves away.

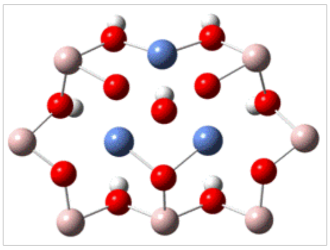

Figure S5. Final optimized geometry of non-linear 3_Al cluster model. GaussView software [3] was used for visualization of the cluster structures.

Following the same line of reasoning for the smallest cluster, we tried also to optimize the non-linear cluster containing 3 Ni atoms, 3_Al, using two and three layers. Again, because of the presence of hydrogen interactions in between, we anticipated that the layers may keep the original structure of the cluster model. As done before, we simplified the optimization, fixing all the atoms except that of the active site. However, the calculation did not reach the geometrical convergence criteria. In fact some of the not fixed atoms move away as seen in Figure S6.

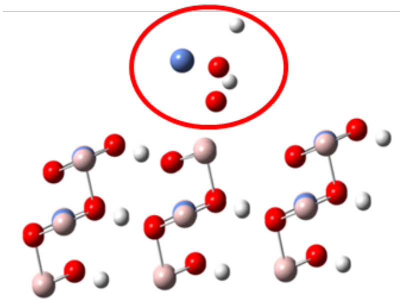

Figure S6. Partially relaxed geometry of the three layers non-linear 3_Al cluster model. GaussView software [3] was used for visualization of the cluster structures.

We got no better results when all the atoms of the first and third layers were described by the ECPs. In this case, the hydrogen atoms that were not fixed fell into the oxygen atoms of the lower layer and the calculations ended with non-convergence error (see Figure S7).

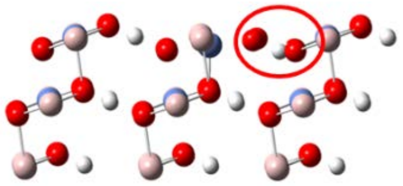

Figure S7. Partially relaxed geometry of the three layers non-linear 3_Al cluster model using ECPs to describe all the atoms of the model except the active site. GaussView software [3] was used for visualization of the cluster structures.

Increasing further the number of atoms of the cluster doesn't help. The non-linear cluster with 4 Ni atoms, 4_Al, in which only the Al atoms are fixed, leads to the same results of the smaller clusters. Also in this case, we need to fix also the H atoms in order to optimize to a reasonable structure.

The biggest cluster we investigated contained 12 Ni atoms. Despite the bigger size of this cluster with respect to the previous ones, the geometry optimization was similar. Optimizing the cluster fixing just Al atoms led to a distortion of the initial geometry. An intermediate geometry showed a "bent" structure (see Figure S8). The calculation continued and ended with error.

Also in this case, it is possible to optimize the geometry only fixing Al and H atoms.

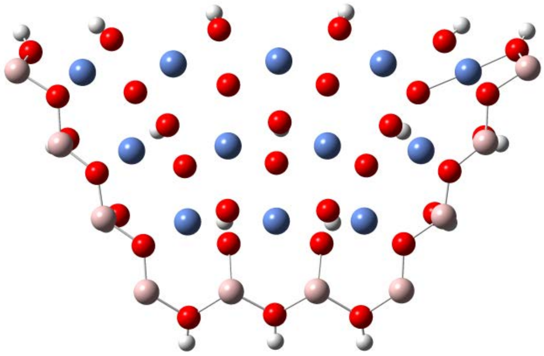

Figure S8. Bent partially relaxed geometry of cluster model 12_Al. GaussView software [3] was used for visualization of the cluster structures.

All the calculations discussed so far have shown that when Al ECPs are used, we are not able to optimize the structure of the smaller clusters 1_Al and 3_Al, while it is possible to optimize the structures of all the bigger clusters non-linear 3_Al, 4_Al, 6_Al and 12_Al, if the Al ECPs atoms and all the hydrogen atoms of the cluster models are kept fixed. However, when the water molecule is added to the bigger clusters in order to simulate the catalysis, the water molecule is not kept coordinated to the Ni atom. This makes the cluster models discussed so far no suitable for investigating catalysis.

Mixed ECPs

As discussed before, theoretical and experimental findings have shown different oxidation states for the Ni atoms of β-NiOOH [5-8]. For this reason, mixed ECPs calculations have been performed using 3_Al cluster model. This cluster model, indeed, represents a good compromise between cluster size and calculation time. The first attempt is to replace Ni atoms with ECPs of Mg^{2+}, Al^{3+} and Si^{4+} that should mimic the three different oxidation states obtained in consequence of the dissociation of Ni^{3+} into Ni^{2+} and Ni^{4+} (see Figure S9, a). However, even in this case the calculation didn't reach the convergence criteria and the structure of the cluster collapsed (see Figure S9b).

Considering an average oxidation state of 2.7, as in [5], we replaced all the Al ECPs except one with four Mg ECPs (see Figure S9, c). In this case the calculation ended reaching the convergence criteria but in the last optimized structure we can see from Figure S9d that the central Ni2 atom of the cluster is out of the plane formed by the other two Ni1 and Ni3 atoms, and the bond distances Ni-O are much longer.

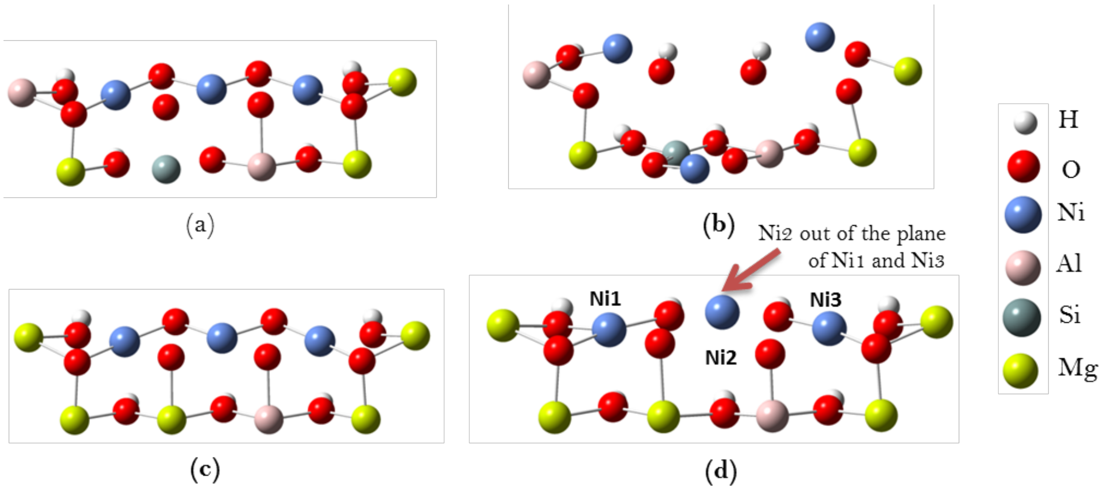

Figure S9. Mixed ECPs cluster models: (**a**) starting geometry and (**b**) final geometry for cluster $3\_Al_2\_Si\_Mg_3$; (**c**) starting geometry and (**d**) final geometry for cluster model $3\_Al\_Mg_4$. GaussView software [3] was used for visualization of the cluster structures.

Cartesian Coordinates (Å) of Initial, Partially Relaxed and Final Optimized Geometries of Cluster Model b)

Initial geometry of cluster model 1_Al

O	-3.45146000	2.98903000	5.62170000
H	-4.31431000	1.49453000	8.67981000
H	-4.31431000	4.48353000	8.67981000
O	-4.31431000	1.49453000	7.58460000
O	-4.31431000	4.48353000	7.58460000
Ni	-5.17716000	2.98903000	6.59443000

O	-6.04001000	1.49453000	5.62170000
H	-6.90286000	2.98903000	8.67981000
O	-6.90286000	2.98903000	7.58460000
Al	-2.58861000	1.49453000	6.59443000
Al	-5.17716000	0.00003300	6.59443000
Al	-7.76571000	1.49453000	6.59443000
Al	-2.58861000	4.48353000	6.59443000

Partially relaxed geometry of cluster model 1_Al

O	-3.43127800	2.96056600	6.62079400
H	-3.03500900	-0.72007700	7.65559200
H	-4.46665000	6.19424100	7.68217200
O	-3.44426700	-0.01052300	7.06284500
O	-4.14788900	5.29003500	7.35756000
Ni	-5.60343000	3.74111300	7.33749700
O	-6.02844100	1.46476400	6.64066700
H	-8.33361900	4.01169500	7.62380900
O	-7.70516000	3.27513100	7.31987900
Al	-2.59849300	1.49322000	6.59345400
Al	-5.17076300	0.00728700	6.59540600
Al	-7.75825900	1.49504000	6.59394100
Al	-2.59257500	4.47707600	6.59491900

Initial geometry of three layers cluster model 1_Al

H	7.76565000	4.48350000	-13.40210000
H	10.35420000	2.98900000	-13.40210000
H	6.03983000	7.47250000	-4.56903000
H	8.62838000	5.97800000	-4.56903000
H	6.90274000	5.97810000	-8.98548000
H	9.49129000	4.48360000	-8.98548000
O	7.76565000	4.48350000	-12.04410000
O	8.62838000	2.98900000	-16.45960000
O	6.90274000	5.97810000	-7.62715000
O	7.76565000	4.48350000	-14.49700000
O	10.35420000	2.98900000	-14.49700000
O	6.03983000	7.47250000	-5.66438000
O	8.62838000	5.97800000	-5.66438000
O	6.90274000	5.97810000	-10.08080000
O	9.49129000	4.48360000	-10.08080000
Ni	8.62838000	5.97800000	-11.07100000
Ni	9.49129000	4.48360000	-15.48710000
Ni	7.76565000	7.47250000	-6.65461000
H	10.35420000	5.97800000	-13.40210000

H	8.62838000	8.96700000	-4.56903000
H	9.49129000	7.47260000	-8.98548000
O	10.35420000	5.97800000	-12.04410000
O	11.21690000	4.48350000	-16.45960000
O	9.49129000	7.47260000	-7.62715000
O	10.35420000	5.97800000	-14.49700000
O	8.62838000	8.96700000	-5.66438000
O	9.49129000	7.47260000	-10.08080000
Al	6.90274000	2.98910000	-15.48710000
Al	12.07980000	5.97810000	-15.48710000
Al	11.21690000	7.47250000	-11.07100000
Al	6.03983000	4.48350000	-11.07100000
Al	10.35420000	8.96700000	-6.65461000
Al	5.17710000	5.97800000	-6.65461000
Al	12.07980000	2.98910000	-15.48710000
Al	9.49129000	1.49460000	-15.48710000
Al	8.62838000	2.98900000	-11.07100000
Al	11.21690000	4.48350000	-11.07100000
Al	7.76565000	4.48350000	-6.65461000
Al	10.35420000	5.97800000	-6.65461000

Partially relaxed geometry of three layers cluster model 1_Al

H	7.75856100	4.48807900	-13.39799200
H	10.35387300	2.98932900	-13.40215000
H	6.03863300	7.47231400	-4.56885800
H	8.62738100	5.97924700	-4.56864000
H	6.23501100	6.52365600	-9.85436600
H	9.48880200	4.48835800	-8.98291200
O	7.38407500	3.80840100	-11.89440400
O	8.62905400	2.98985700	-16.45964800
O	6.89439800	5.98331400	-7.64258900
O	7.76562700	4.48436100	-14.49707000
O	10.35390400	2.98921300	-14.49740500
O	6.04049400	7.47079100	-5.66271300
O	8.62897800	5.97759900	-5.66295200
O	6.80878100	6.21729800	-10.57091100
O	9.49758200	4.47043700	-10.09635300
Ni	6.82142300	8.96995500	-11.39984800
Ni	9.49535800	4.47670800	-15.48935100
Ni	7.77390600	7.45906200	-6.64884100
H	10.35406200	5.98723400	-13.39786200
H	8.62927700	8.96769700	-4.56889900
H	9.28224300	8.30132800	-9.84986400

O	11.15066100	5.99527000	-11.90397600
O	11.21571600	4.48342400	-16.45962700
O	9.48945700	7.48064600	-7.64206700
O	10.35328500	5.97813100	-14.49687700
O	8.62953600	8.96597100	-5.66269600
O	9.28233400	7.62047000	-10.59988600
Al	6.89955900	2.98669000	-15.48931700
Al	12.08343600	5.97960000	-15.48909500
Al	11.19525500	7.44780300	-11.06392100
Al	6.07118900	4.49110400	-11.06373900
Al	10.34960000	8.97123100	-6.65293900
Al	5.17654500	5.98425200	-6.65306800
Al	12.07944500	2.98820200	-15.48769000
Al	9.49227800	1.49437400	-15.48769700
Al	8.63126800	2.99765000	-11.06711400
Al	11.20801700	4.48764700	-11.06770300
Al	7.75823400	4.47659200	-6.65119000
Al	10.36418900	5.98148300	-6.65088600

Initial geometry of cluster model 3_Al

H	4.48427600	-1.81107500	1.69030800
H	2.98685300	0.74905200	2.06124700
O	4.47946900	0.33398900	-1.09038900
O	4.48325400	-1.65459900	0.60622300
O	2.98583100	0.90552800	0.97716300
Ni	2.98695800	-0.66095600	-0.24940600
H	1.49527900	-1.81481600	1.69258700
H	-0.00214400	0.74531100	2.06352700
O	1.49047200	0.33024800	-1.08811000
O	1.49425700	-1.65834000	0.60850200
O	-0.00316600	0.90178700	0.97944200
Ni	-0.00203900	-0.66469600	-0.24712700
H	-1.49371800	-1.81855700	1.69486600
H	-2.99110600	0.74155100	2.06580300
O	-1.49852500	0.32650800	-1.08583000
O	-1.49474000	-1.66208100	0.61078200
O	-2.99212800	0.89802700	0.98171800
Ni	-2.99101000	-0.66845200	-0.24485000
H	-4.48268000	-1.82231700	1.69714300
O	-4.48747800	0.32274200	-1.08355500
O	-4.48370200	-1.66584100	0.61305800
Al	5.97595500	-0.65721500	-0.25168500
Al	4.47853200	1.90291200	0.11925400

Al	1.48953500	1.89917100	0.12153300
Al	-1.49943600	1.89541600	0.12381000
Al	-4.48847600	1.89170000	0.12609300
Al	-5.92646100	-0.63644600	-0.27276200

Partially relaxed geometry of cluster model 3_Al

H	5.03925400	-2.25900900	1.64968700
H	3.22066600	2.27208500	1.87977400
O	4.85900600	0.37468400	-0.83618400
O	4.64231500	-1.68417500	0.84458800
O	3.16148500	1.98798400	0.90886900
Ni	2.84119300	-1.12116800	-0.16386500
H	1.50567300	-1.76508700	2.28656500
H	0.04916200	2.57215000	1.73482600
O	1.48256900	0.08771300	-0.82011500
O	1.38018200	-1.53028800	1.30926100
O	0.16442100	2.13967800	0.83640500
Ni	-0.12347400	-1.11204000	0.08579500
H	-1.81972300	-1.70091400	2.45018400
H	-3.08146700	2.46386000	1.78329900
O	-1.50587000	0.02721900	-0.78012700
O	-1.76342500	-1.45315200	1.45795000
O	-2.83212500	2.14135900	0.85820300
Ni	-3.18390700	-1.01517200	0.06953400
H	-5.74348800	-1.53929100	1.18575300
O	-4.28764100	0.36375000	-0.96998900
O	-5.33728500	-0.96871700	0.46236700
Al	6.15382000	-0.48962300	-1.30343700
Al	4.65407700	1.74002300	-0.02042500
Al	1.66653200	1.75080900	-0.04845900
Al	-1.32919500	1.75305600	-0.08181800
Al	-4.31727400	1.75157600	-0.09660800
Al	-5.73764000	-0.42719200	-1.44163000

Initial geometry of cluster model Non-linear 3_Al

H	6.90274000	-2.98890000	4.26352000
O	7.76565000	-4.48350000	1.20490000
O	6.90274000	-2.98890000	3.16820000
H	6.90274000	0.00010000	4.26352000
H	9.49129000	-4.48340000	4.26352000
H	9.49129000	-1.49440000	4.26352000
O	7.76565000	-1.49450000	1.20490000
O	10.35420000	-2.98900000	1.20490000

O	6.90274000	0.00010000	3.16820000
O	9.49129000	-4.48340000	3.16820000
O	9.49129000	-1.49440000	3.16820000
Ni	8.62838000	-2.98900000	2.17800000
Ni	8.62838000	0.00000000	2.17800000
H	9.49129000	1.49460000	4.26352000
H	12.07980000	-2.98890000	4.26352000
H	12.07980000	0.00010000	4.26352000
O	10.35420000	0.00000000	1.20490000
O	12.94270000	-1.49450000	1.20490000
O	9.49129000	1.49460000	3.16820000
O	12.07980000	-2.98890000	3.16820000
O	12.07980000	0.00010000	3.16820000
Ni	11.21690000	-1.49450000	2.17800000
Al	13.80550000	0.00000000	2.17800000
Al	13.80550000	-2.98900000	2.17800000
Al	11.21690000	-4.48350000	2.17800000
Al	8.62838000	-5.97800000	2.17800000
Al	6.03983000	-4.48350000	2.17800000
Al	6.03983000	-1.49450000	2.17800000
Al	11.21690000	1.49450000	2.17800000

Final *optimized* geometry of cluster model Non-linear 3_Al

H	6.90259600	-2.98899800	4.26334000
O	7.65976700	-4.67448800	1.72794200
O	6.56767500	-3.00519900	3.22201600
H	6.90275300	-0.00011100	4.26305400
H	9.49133100	-4.48348600	4.26314700
H	9.49138400	-1.49459500	4.26352500
O	7.76136700	-1.38682100	1.38747900
O	10.46518400	-3.18513600	1.33688000
O	7.01865200	-0.33106700	3.32884400
O	9.67908900	-4.81080400	3.25958000
O	9.52533200	-1.55518500	3.27199000
Ni	8.70866100	-2.95933700	2.03542500
Ni	8.56273400	0.11408500	2.35781300
H	9.49148300	1.49447400	4.26304900
H	12.07986400	-2.98898000	4.26314500
H	12.07997300	0.00015500	4.26334300
O	10.26391100	0.05700700	1.38834100
O	13.16033300	-1.49860000	1.72845400
O	9.72104700	1.22913300	3.32915900
O	12.26992300	-3.31585600	3.25954000

O	12.26197100	0.28243400	3.22216500
Ni	11.15029500	-1.55105000	2.03493900
Al	13.80530700	-0.00101200	2.17796900
Al	13.80494300	-2.98799500	2.17792400
Al	11.21703700	-4.48369800	2.17914800
Al	8.62780400	-5.97705900	2.17789800
Al	6.04079900	-4.48386700	2.17795300
Al	6.04027200	-1.49396900	2.17859400
Al	11.21624300	1.49434100	2.17855100

Initial geometry of three layers cluster model Non-linear 3_Al

H	7.76565000	1.49450000	-13.40210000
H	6.03983000	4.48350000	-4.56903000
H	6.90274000	2.98910000	-8.98548000
O	7.76565000	1.49450000	-12.04410000
O	8.62838000	0.00000000	-16.45960000
O	6.90274000	2.98910000	-7.62715000
O	7.76565000	1.49450000	-14.49700000
O	6.03983000	4.48350000	-5.66438000
O	6.90274000	2.98910000	-10.08080000
H	7.76565000	4.48350000	-13.40210000
H	10.35420000	0.00000000	-13.40210000
H	10.35420000	2.98900000	-13.40210000
H	6.03983000	7.47250000	-4.56903000
H	8.62838000	2.98900000	-4.56903000
H	8.62838000	5.97800000	-4.56903000
H	6.90944800	5.97900500	-8.99718200
H	9.49129000	1.49460000	-8.98548000
H	9.49129000	4.48360000	-8.98548000
O	7.75250700	4.47694600	-12.04613300
O	10.35420000	2.98900000	-12.04410000
O	8.62838000	2.98900000	-16.45960000
O	11.21690000	1.49450000	-16.45960000
O	6.90274000	5.97810000	-7.62715000
O	9.49129000	4.48360000	-7.62715000
O	7.76565000	4.48350000	-14.49700000
O	10.35420000	0.00000000	-14.49700000
O	10.35420000	2.98900000	-14.49700000
O	6.03983000	7.47250000	-5.66438000
O	8.62838000	2.98900000	-5.66438000
O	8.62838000	5.97800000	-5.66438000
O	6.90328100	5.96469000	-10.08550800
O	9.49129000	1.49460000	-10.08080000

O	9.49129000	4.48360000	-10.08080000
Ni	8.62838000	2.98900000	-11.07100000
Ni	8.62473300	5.98387600	-11.07557300
Ni	9.49129000	1.49460000	-15.48710000
Ni	9.49129000	4.48360000	-15.48710000
Ni	7.76565000	4.48350000	-6.65461000
Ni	7.76565000	7.47250000	-6.65461000
H	10.35420000	5.97800000	-13.40210000
H	12.94270000	1.49450000	-13.40210000
H	12.94270000	4.48350000	-13.40210000
H	8.62838000	8.96700000	-4.56903000
H	11.21690000	4.48350000	-4.56903000
H	11.21690000	7.47250000	-4.56903000
H	9.48562400	7.46888100	-8.99500400
H	12.07980000	2.98910000	-8.98548000
H	12.07980000	5.97810000	-8.98548000
O	10.36638600	5.98579000	-12.04541000
O	12.94270000	4.48350000	-12.04410000
O	11.21690000	4.48350000	-16.45960000
O	13.80550000	2.98900000	-16.45960000
O	9.49129000	7.47260000	-7.62715000
O	12.07980000	5.97810000	-7.62715000
O	10.35420000	5.97800000	-14.49700000
O	12.94270000	1.49450000	-14.49700000
O	12.94270000	4.48350000	-14.49700000
O	8.62838000	8.96700000	-5.66438000
O	11.21690000	4.48350000	-5.66438000
O	11.21690000	7.47250000	-5.66438000
O	9.50151200	7.46624700	-10.08519600
O	12.07980000	2.98910000	-10.08080000
O	12.07980000	5.97810000	-10.08080000
Ni	11.21690000	4.48350000	-11.07100000
Ni	12.07980000	2.98910000	-15.48710000
Ni	10.35420000	5.97800000	-6.65461000
Al	6.90274000	2.98910000	-15.48710000
Al	12.07980000	5.97810000	-15.48710000
Al	14.66840000	4.48360000	-15.48710000
Al	6.90274000	0.00010000	-15.48710000
Al	9.49129000	-1.49440000	-15.48710000
Al	12.07980000	0.00010000	-15.48710000
Al	14.66840000	1.49460000	-15.48710000
Al	11.21690000	7.47250000	-11.07100000
Al	6.03983000	4.48350000	-11.07100000

Al	6.03983000	1.49450000	-11.07100000
Al	13.80550000	5.97800000	-11.07100000
Al	13.80550000	2.98900000	-11.07100000
Al	11.21690000	1.49450000	-11.07100000
Al	8.62838000	0.00000000	-11.07100000
Al	10.35420000	8.96700000	-6.65461000
Al	5.17710000	5.97800000	-6.65461000
Al	5.17710000	2.98900000	-6.65461000
Al	12.94270000	7.47250000	-6.65461000
Al	12.94270000	4.48350000	-6.65461000
Al	10.35420000	2.98900000	-6.65461000
Al	7.76565000	1.49450000	-6.65461000

Partially relaxed geometry of three layers cluster model Non-linear 3_Al

H	7.76547000	1.49415000	-13.40196700
H	6.03972700	4.48313100	-4.56908500
H	6.90257200	2.98895200	-8.98614000
O	7.76583100	1.49405300	-12.04318100
O	8.62844300	0.00003500	-16.45960900
O	6.90270900	2.98933100	-7.62721600
O	7.76554800	1.49434000	-14.49698800
O	6.03984000	4.48384100	-5.66374000
O	6.90073100	2.98833400	-10.08087700
H	7.76564800	4.48362100	-13.40236200
H	10.35420100	0.00000300	-13.40209800
H	10.35419700	2.98911000	-13.40208600
H	6.04009800	7.47246700	-4.56860000
H	8.62834400	2.98891900	-4.56899900
H	8.62863600	5.97782400	-4.56894300
H	3.93335100	9.16546400	-11.37957000
H	9.49134300	1.49480500	-8.98554400
H	9.49111900	4.48382800	-8.98541000
O	7.38559800	4.40676000	-12.14596500
O	10.35443000	2.98871000	-12.04437600
O	8.62831600	2.98908200	-16.45956400
O	11.21687600	1.49456100	-16.45958400
O	6.90536600	5.97548700	-7.63214100
O	9.49113400	4.48416400	-7.62678100
O	7.76570400	4.48333100	-14.49740900
O	10.35421400	0.00001900	-14.49699300
O	10.35418800	2.98906200	-14.49696400
O	6.03904600	7.47270400	-5.66418400
O	8.62828700	2.98910600	-5.66428100

O	8.62805900	5.97854500	-5.66301200
O	5.71876200	8.12338200	-11.55768500
O	9.49117200	1.49467600	-10.08091100
O	9.49110700	4.48259200	-10.08180500
Ni	8.62841700	2.98878900	-11.07172500
Ni	7.76489000	9.03308900	-12.97983600
Ni	9.49127000	1.49467200	-15.48707600
Ni	9.49147900	4.48319100	-15.48719700
Ni	7.76522600	4.48401100	-6.65391200
Ni	7.76477400	7.47356000	-6.65447000
H	10.35409700	5.97885900	-13.40235600
H	12.94270100	1.49449900	-13.40209900
H	12.94285500	4.48344900	-13.40204800
H	8.62802800	8.96713500	-4.56871800
H	11.21688400	4.48344300	-4.56902500
H	11.21706600	7.47245200	-4.56908700
H	9.37208200	9.66137800	-10.94456700
H	12.07980000	2.98912700	-8.98552400
H	12.08024500	5.97833000	-8.98553200
O	10.80529300	6.20533800	-12.17637100
O	12.94297600	4.48339000	-12.04379500
O	11.21685000	4.48359100	-16.45955300
O	13.80546200	2.98898100	-16.45960500
O	9.49069800	7.46980000	-7.62990300
O	12.07976800	5.97827000	-7.62720200
O	10.35431500	5.97788600	-14.49722800
O	12.94268400	1.49450400	-14.49699400
O	12.94277800	4.48348600	-14.49697200
O	8.62917600	8.96729200	-5.66421800
O	11.21689000	4.48358400	-5.66432800
O	11.21694300	7.47265600	-5.66419300
O	9.40321200	8.65567700	-11.22169600
O	12.07988400	2.98931100	-10.08089900
O	12.08015200	5.97827900	-10.08113100
Ni	11.21646600	4.48314700	-11.07129000
Ni	12.07975700	2.98916000	-15.48707900
Ni	10.35454700	5.97883300	-6.65407700
Al	6.90237400	2.98911000	-15.48723400
Al	12.07992100	5.97830100	-15.48715300
Al	14.66844200	4.48351400	-15.48708300
Al	6.90280200	-0.00000900	-15.48713800
Al	9.49135300	-1.49441300	-15.48712800
Al	12.07979900	0.00011700	-15.48709800

Al	14.66839000	1.49455400	-15.48712000
Al	11.21494800	7.47227100	-11.06840100
Al	6.04127900	4.48712900	-11.06545400
Al	6.04138100	1.49346800	-11.07465200
Al	13.80588200	5.97749800	-11.07237100
Al	13.80560000	2.98900800	-11.07059300
Al	11.21688400	1.49454600	-11.07084000
Al	8.62814100	-0.00011500	-11.06970400
Al	10.35413200	8.96721000	-6.65394500
Al	5.17659700	5.97857100	-6.65403200
Al	5.17731500	2.98912600	-6.65425200
Al	12.94260500	7.47248400	-6.65445900
Al	12.94257000	4.48339700	-6.65478700
Al	10.35415000	2.98918800	-6.65467400
Al	7.76581900	1.49457500	-6.65492100

Initial geometry of cluster model 4_Al

H	5.17710000	2.98900000	-0.15310000
O	5.17710000	2.98900000	-1.24800000
O	6.03980000	1.49450000	-3.21060000
O	6.03980000	-1.49450000	-3.21060000
H	5.17710000	0.00000000	-0.15310000
O	5.17710000	0.00000000	-1.24800000
H	7.76560000	4.48350000	-0.15310000
O	7.76560000	4.48350000	-1.24800000
Ni	6.90270000	2.98910000	-2.23810000
O	8.62840000	2.98900000	-3.21060000
Ni	9.49130000	-1.49440000	-2.23810000
H	10.35420000	-2.98900000	-0.15310000
O	8.62840000	-2.98900000	-3.21060000
O	10.35420000	-2.98900000	-1.24800000
Ni	6.90270000	0.00010000	-2.23810000
H	7.76560000	-1.49450000	-0.15310000
O	8.62840000	0.00000000	-3.21060000
O	7.76560000	-1.49450000	-1.24800000
H	7.76560000	1.49450000	-0.15310000
O	7.76560000	1.49450000	-1.24800000
O	11.21690000	-1.49450000	-3.21060000
Ni	9.49130000	1.49460000	-2.23810000
H	10.35420000	0.00000000	-0.15310000
O	11.21690000	1.49450000	-3.21060000
O	10.35420000	0.00000000	-1.24800000
H	10.35420000	2.98900000	-0.15310000

O	10.35420000	2.98900000	-1.24800000
Al	9.49130000	4.48360000	-2.23810000
Al	4.31420000	1.49460000	-2.23810000
Al	4.31420000	-1.49440000	-2.23810000
Al	12.07980000	2.98910000	-2.23810000
Al	12.07980000	0.00010000	-2.23810000
Al	6.90270000	-2.98890000	-2.23810000
Al	9.49130000	-4.48340000	-2.23810000
Al	12.07980000	-2.98890000	-2.23810000

Final *optimized* geometry of cluster model 4_Al

H	5.17710400	2.98897200	-0.15319000
O	5.30756700	2.62692100	-1.07481900
O	6.08006200	1.49211600	-2.98594500
O	5.88796800	-1.73106300	-2.74206900
H	5.17711200	0.00003000	-0.15314900
O	4.84143600	-0.02898400	-1.18333300
H	7.76562500	4.48346600	-0.15319400
O	8.01378300	4.19104600	-1.07499900
Ni	6.88241000	3.02388600	-2.03403800
O	8.60884400	2.95683200	-2.98471500
Ni	9.50986200	-1.52864300	-2.11803600
H	10.35418700	-2.98896800	-0.15312700
O	8.51733000	-3.22494100	-2.72934200
O	10.53060100	-3.29378700	-1.15787900
Ni	6.97598800	-0.08252900	-2.17760900
H	7.76557000	-1.49466200	-0.15320600
O	8.68814600	-0.10559900	-3.15013500
O	7.71528700	-1.65907100	-1.09764800
H	7.76566000	1.49443400	-0.15318500
O	7.81316100	1.41354200	-1.13104100
O	11.47605200	-1.51608100	-2.72931400
Ni	9.52617100	1.38971500	-2.18510000
H	10.35437200	-0.00003900	-0.15322400
O	11.49870800	1.50772000	-2.74233400
O	10.51957300	-0.03827400	-1.09872600
H	10.35417400	2.98900600	-0.15314400
O	10.54811300	3.26703000	-1.18331600
Al	9.49093000	4.48367800	-2.23785000
Al	4.31432700	1.49500300	-2.23787000
Al	4.31419600	-1.49506600	-2.23852900
Al	12.08033700	2.98876800	-2.23859400
Al	12.07880300	0.00014500	-2.23726200

Al	6.90314400	-2.98809100	-2.23745100
Al	9.49062400	-4.48382500	-2.23824000
Al	12.08053500	-2.98855100	-2.23838400

Initial geometry of cluster model 12_Al

H	-1.72582000	14.94500000	-4.56903000
O	-0.86290800	13.45060000	-7.62715000
O	-1.72582000	14.94500000	-5.66438000
Al	-2.58855000	13.45050000	-6.65461000
Al	0.00000000	11.95600000	-6.65461000
H	0.86273500	13.45050000	-4.56903000
H	0.86273500	16.43950000	-4.56903000
H	3.45128000	11.95600000	-4.56903000
O	1.72564000	11.95610000	-7.62715000
O	1.72564000	14.94510000	-7.62715000
O	0.86273500	13.45050000	-5.66438000
O	0.86273500	16.43950000	-5.66438000
O	3.45128000	11.95600000	-5.66438000
Ni	0.00000000	14.94500000	-6.65461000
Al	2.58855000	10.46150000	-6.65461000
Ni	2.58855000	13.45050000	-6.65461000
H	6.03983000	10.46150000	-4.56903000
H	3.45128000	14.94500000	-4.56903000
H	3.45128000	17.93400000	-4.56903000
H	6.03983000	13.45050000	-4.56903000
O	4.31419000	13.45060000	-7.62715000
O	4.31419000	16.43960000	-7.62715000
O	4.31419000	10.46160000	-7.62715000
O	6.03983000	10.46150000	-5.66438000
O	3.45128000	14.94500000	-5.66438000
O	3.45128000	17.93400000	-5.66438000
O	6.03983000	13.45050000	-5.66438000
Al	5.17710000	8.96700000	-6.65461000
Ni	2.58855000	16.43950000	-6.65461000
Ni	5.17710000	11.95600000	-6.65461000
Ni	5.17710000	14.94500000	-6.65461000
H	8.62838000	11.95600000	-4.56903000
H	6.03983000	16.43950000	-4.56903000
H	6.03983000	19.42850000	-4.56903000
H	8.62838000	14.94500000	-4.56903000
O	6.90274000	14.94510000	-7.62715000
O	6.90274000	17.93410000	-7.62715000
O	6.90274000	11.95610000	-7.62715000

O	8.62838000	11.95600000	-5.66438000
O	6.03983000	16.43950000	-5.66438000
O	6.03983000	19.42850000	-5.66438000
O	8.62838000	14.94500000	-5.66438000
Al	7.76565000	10.46150000	-6.65461000
Ni	5.17710000	17.93400000	-6.65461000
Ni	7.76565000	13.45050000	-6.65461000
Ni	7.76565000	16.43950000	-6.65461000
H	11.21690000	13.45050000	-4.56903000
H	8.62838000	17.93400000	-4.56903000
H	8.62838000	20.92300000	-4.56903000
H	11.21690000	16.43950000	-4.56903000
O	9.49129000	16.43960000	-7.62715000
O	9.49129000	19.42860000	-7.62715000
O	9.49129000	13.45060000	-7.62715000
O	11.21690000	13.45050000	-5.66438000
O	8.62838000	17.93400000	-5.66438000
O	8.62838000	20.92300000	-5.66438000
O	11.21690000	16.43950000	-5.66438000
Al	10.35420000	11.95600000	-6.65461000
Ni	7.76565000	19.42850000	-6.65461000
Ni	10.35420000	14.94500000	-6.65461000
Ni	10.35420000	17.93400000	-6.65461000
H	11.21690000	19.42850000	-4.56903000
H	11.21690000	22.41750000	-4.56903000
O	12.07980000	17.93410000	-7.62715000
O	12.07980000	20.92310000	-7.62715000
O	12.07980000	14.94510000	-7.62715000
O	11.21690000	19.42850000	-5.66438000
O	11.21690000	22.41750000	-5.66438000
Al	12.94270000	13.45050000	-6.65461000
Ni	10.35420000	20.92300000	-6.65461000
Al	12.94270000	16.43950000	-6.65461000
Al	12.94270000	19.42850000	-6.65461000
Al	12.94270000	22.41750000	-6.65461000

Bent partially relaxed geometry of cluster model 12_Al

H	-3.08620700	14.57617900	-4.22728900
O	-0.90672700	13.38043100	-6.58637900
O	-2.48542500	14.18533200	-4.91472400
Al	-2.58929400	13.45094000	-6.64773500
Al	0.00838900	11.96133400	-6.66097700
H	-0.27459200	11.64830700	-3.89358700

H	-1.12621700	15.89920800	-2.65532200
H	2.68927300	10.17942900	-3.91946800
O	1.60327300	11.72573200	-6.72989000
O	1.27289800	14.65326900	-4.71157800
O	0.06302400	12.13231800	-4.67837600
O	-0.35823900	15.73318700	-3.29466000
O	2.91564100	10.69889800	-4.71593400
Ni	-0.60962600	14.38998500	-4.68877700
Al	2.59442600	10.46306100	-6.66187800
Ni	2.22173700	12.85013700	-4.59823100
H	7.26745200	8.06993400	-5.72613600
H	2.57062500	13.75034900	-1.93100600
H	2.23990400	18.34402000	-0.93004700
H	6.27646400	12.24261800	-2.61987700
O	4.12103800	13.36681800	-4.97372000
O	3.94573000	16.49568100	-3.50829800
O	4.29500500	10.34264700	-6.57388600
O	6.90370400	8.89602000	-6.16125200
O	2.78990600	14.13905700	-2.81049300
O	2.67983100	17.96502400	-1.76411600
O	6.16089200	12.68180300	-3.48826300
Al	5.18322500	8.97248300	-6.63399400
Ni	1.89645600	16.14695100	-3.20118700
Ni	5.16966000	11.73287700	-4.99222400
Ni	5.03548000	14.65051600	-3.44184500
H	9.94470800	9.67792700	-5.87129400
H	6.25628200	15.99634900	-1.15060800
H	6.25081400	20.65270600	-0.93810000
H	9.53167100	14.18439800	-2.61030100
O	6.82621400	15.03758400	-4.48959800
O	6.95576800	18.24496800	-3.51676600
O	6.80054400	11.80824200	-6.06540200
O	9.47432700	10.49090500	-6.20616900
O	6.13210900	16.20527000	-2.10170700
O	6.34507900	20.07434400	-1.76857300
O	9.20618900	14.46253300	-3.49160000
Al	7.75575700	10.45458100	-6.66793200
Ni	5.05412100	18.06876500	-2.54641000
Ni	7.84720100	13.28659000	-4.78227900
Ni	8.04836800	16.41012800	-3.44867300
H	12.66426100	11.20423800	-5.71073100
H	9.98267900	18.11117500	-1.90023700
H	9.94513900	22.37411700	-2.49767600

H	13.10475800	16.21118900	-3.92154600
O	9.63905200	16.56175100	-4.95116200
O	9.83902400	19.67599400	-4.69853600
O	9.62234400	13.44833600	-6.05235100
O	12.13334400	11.92907500	-6.15478700
O	9.54828700	18.10538000	-2.78227200
O	9.70164600	21.69132700	-3.21790500
O	12.54827700	16.26179900	-4.72254600
Al	10.36465200	11.96183000	-6.67014000
Ni	8.27136900	19.85538500	-3.17864100
Ni	10.53797300	14.83756400	-4.99345100
Ni	10.99523500	17.97261300	-4.62380500
H	13.31266400	19.50472000	-3.91386800
H	12.26196300	23.48077900	-4.29949800
O	12.32777900	17.92053000	-6.73825500
O	12.16690400	20.92930800	-6.63946900
O	12.21844200	14.90282300	-6.57981500
O	12.72908800	19.44895300	-4.69792700
O	12.29125200	22.74223200	-4.95384300
Al	12.93912700	13.44918800	-6.63352700
Ni	11.09009300	21.21313200	-4.73816400
Al	12.93328600	16.43154300	-6.65890500
Al	12.93513600	19.42427900	-6.66367100
Al	12.94304600	22.41926100	-6.64734000

Cartesian coordinates (Å) of initial, partially relaxed and final optimized geometries of mixed ECPs clusters

Initial geometry of 3_Al_2_Si_Mg_3

H	4.50674800	-1.91686200	1.49212300
H	3.00918700	0.63820700	1.89709900
O	4.42647500	0.26372700	-1.05046100
O	4.57885400	-1.19617000	0.85734000
O	3.02471800	1.17832600	1.07712000
Ni	3.03865700	-0.66313900	-0.16523400
H	1.51772600	-1.92114000	1.49748700
H	0.02024600	0.63384900	1.90249400
O	1.51125600	0.12892200	-1.06269000
O	1.54722000	-1.54419900	0.60842100
O	0.01985700	1.12499800	1.05198400
Ni	0.03097900	-0.75443400	-0.21441500
H	-1.47123600	-1.92548700	1.50286100
H	-2.96866500	0.62949000	1.90783800
O	-1.45329900	0.12366400	-1.05732300

O	-1.48497100	-1.55128300	0.61225100
O	-2.98487400	1.17245200	1.09010800
Ni	-2.97098800	-0.66853300	-0.15796400
H	-4.46021900	-1.92992800	1.50824300
O	-4.33913400	0.27056000	-1.05044800
O	-4.51695300	-1.20763600	0.86888800
Mg	-4.46817300	1.80370700	-0.01530600
Mg	4.49856200	1.81673900	-0.03143100
Si	-1.48009800	1.80747100	-0.02077600
Al	-5.90605400	-0.71933000	-0.44495100
Al	1.51043400	1.81181400	-0.02620500
Mg	5.99589100	-0.73802700	-0.43664800

Partially relaxed geometry of 3_Al_2_Si_Mg_3

H	4.44997200	-0.55822300	1.91441800
H	3.01749900	2.05989800	1.70372300
O	4.43356700	0.56787500	-0.77686300
O	4.92599400	-0.33952300	1.06715700
O	2.71398300	1.83054400	0.71544400
Ni	2.98095800	-1.06756500	-0.11539000
H	1.46130600	-0.48347900	1.94517200
H	0.02980500	2.13063200	1.73862200
O	0.95872800	-0.21830400	-0.40393400
O	1.41701000	-1.27367200	1.29381000
O	-0.09252500	2.49858000	0.37533500
Ni	0.24645700	-3.77624100	-0.41452400
H	-1.52628000	-0.41210300	1.97954800
H	-2.95971800	2.20384600	1.77372400
O	-1.31223500	-0.94731000	0.04970400
O	-1.43663200	-1.40252200	1.23281600
O	-2.67349700	2.38793600	0.65910900
Ni	-3.48275600	-1.59168200	-0.30431200
H	-4.51584300	-0.33872400	2.01362900
O	-4.14707900	0.60342400	-0.70014100
O	-4.53868000	-0.46916300	1.04215600
Mg	-4.45258800	2.91467400	-0.35588400
Mg	4.49329000	2.69285100	-0.45285800
Si	-1.48105800	2.85233100	-0.38958600
Si	5.93524100	0.08615400	-0.25411400
Al	-5.95868000	0.40818500	-0.15901200
Al	1.52458300	2.76564600	-0.41256900

Initial geometry of 3_Al_Mg_5

H	4.46914500	-1.90917900	1.49257200
H	2.97241800	0.64617000	1.89885700
O	4.39247500	0.27424200	-1.04769200
O	4.54223700	-1.18782500	0.85865400
O	2.98903200	1.18718200	1.07948900
Ni	3.00332300	-0.65292200	-0.16487400
H	1.48011900	-1.91201100	1.49493900
H	-0.01652900	0.64325800	1.90125600
O	1.47720600	0.14086600	-1.06298800
O	1.51068600	-1.53410700	0.60631800
O	-0.01582800	1.13534100	1.05128700
Ni	-0.00434800	-0.74270300	-0.21716600
H	-1.50885000	-1.91491300	1.49731600
H	-3.00544500	0.64034300	1.90360300
O	-1.48735600	0.13704100	-1.06059300
O	-1.52151000	-1.53972400	0.60710600
O	-3.02057200	1.18421300	1.08645500
Ni	-3.00632800	-0.65540700	-0.16362600
H	-4.49783800	-1.91790900	1.49970200
O	-4.37312500	0.28533100	-1.05644600
O	-4.55358200	-1.19488600	0.86108500
Mg	-4.50245700	1.81740100	-0.01974900
Mg	4.46429500	1.82609800	-0.02688400
Al	1.47616100	1.82261800	-0.02465400
Mg	5.96079100	-0.72894800	-0.43341000
Mg	-5.94112900	-0.70446300	-0.45360700
Mg	-1.51437700	1.81972100	-0.02222300

Final *optimized* geometry of 3_Al_Mg_5

H	4.46100000	-1.84100200	1.61416900
H	2.97354300	0.73641200	1.89556300
O	4.37358700	0.20084500	-1.01688900
O	4.51038200	-1.10127300	0.97964900
O	2.90991500	1.30090100	1.02925900
Ni	2.99455200	-0.66134800	-0.12622500
H	1.47218800	-1.83271200	1.61409900
H	-0.01539100	0.74466600	1.89582500
O	1.45968600	0.34312700	-1.00309600
O	1.50441600	-1.30715000	0.80845700
O	0.06916600	1.37410400	1.04770900
Ni	0.07145100	-1.02998800	-0.46646100
H	-1.51706300	-1.82444900	1.61431600
H	-3.00436600	0.75297400	1.89599300

O	-1.56520300	0.06116400	-1.09821000
O	-1.49178200	-1.32993900	0.77302000
O	-2.99735800	1.19647400	1.01780800
Ni	-3.05844300	-0.66510500	-0.07100800
H	-4.50592500	-1.81644400	1.61453600
O	-4.34978500	0.23507400	-1.03849200
O	-4.56566600	-1.10963100	0.95738100
Mg	-4.49556000	1.84138500	-0.08297500
Mg	4.47075700	1.81673000	-0.08327100
Al	1.48309000	1.82428600	-0.08308500
Mg	5.95813300	-0.76054100	-0.36526100
Mg	-5.94345600	-0.69327100	-0.39616400
Mg	-1.50776500	1.83199900	-0.08347300

SI-3. Cartesian Coordinates (Å) of Optimized Geometries of Mg ECPs Cluster Models

Final *optimized* geometry of 3_Mg

HF (Hartree atomic energy) =-1341.096833

H	4.47711000	-1.89674000	1.49281400
H	2.97761400	0.65706700	1.89858000
O	4.39803400	0.28605900	-1.04791100
O	4.54941000	-1.17544100	0.85874300
O	2.99362900	1.19792400	1.07909700
Ni	3.00990100	-0.64242700	-0.16487700
H	1.48808800	-1.90281900	1.49522300
H	-0.01132800	0.65090900	1.90102000
O	1.48291100	0.14951300	-1.06313700
O	1.51823200	-1.52507000	0.60652200
O	-0.01117400	1.14281400	1.05094600
Ni	0.00232900	-0.73548500	-0.21710900
H	-1.50087500	-1.90896700	1.49764200
H	-3.00024000	0.64474900	1.90340900
O	-1.48164400	0.14246900	-1.06070200
O	-1.51395600	-1.53397900	0.60735200
O	-3.01596900	1.18842800	1.08614600
Ni	-2.99974300	-0.65143800	-0.16354600
H	-4.48985800	-1.91520800	1.50006900
O	-4.36757300	0.28762500	-1.05654600
O	-4.54639600	-1.19238200	0.86130000
Mg	-5.93449300	-0.70374400	-0.45347600
Mg	-4.49855600	1.81977400	-0.02017100
Mg	-1.51048000	1.82533800	-0.02268700
Mg	1.48005300	1.83148300	-0.02516000
Mg	4.46818100	1.83820800	-0.02743200

Mg	5.96744700	-0.71529700	-0.43343800

Final *optimized* geometry of 3_Mg+H_2O

HF=-1417.5375924

H	4.47664600	-1.46609400	1.88887700
H	2.97691100	1.11849200	1.81268200
O	4.41528100	0.21244800	-1.02075300
O	4.55614800	-0.88789200	1.12568300
O	3.00240900	1.49146600	0.90535900
Ni	3.01950100	-0.53135900	0.02794200
H	1.48753100	-1.47189500	1.89244300
H	-0.01202200	1.11261200	1.81617900
O	1.52274800	0.07218100	-1.03440100
O	1.54195200	-1.27999700	0.94513100
O	-0.01159800	1.50243100	0.90699900
Ni	0.00295700	-0.81196100	-0.14067600
H	-1.50121600	-1.47774800	1.89606300
H	-3.00093100	1.10669400	1.81995100
O	-1.51809400	0.06670500	-1.03106300
O	-1.53649500	-1.28387600	0.94887500
O	-3.02646200	1.48323900	0.91422900
Ni	-3.00861200	-0.54055600	0.02980400
H	-4.49028100	-1.48377700	1.89978500
O	-4.38632200	0.21105200	-1.03082500
O	-4.55380500	-0.90431300	1.13165400
Mg	-5.93502200	-0.65696400	-0.24493000
Mg	-4.49934700	1.90327500	-0.28858500
Mg	-1.51117500	1.90851200	-0.29199500
Mg	1.47905400	1.91440500	-0.29570200
Mg	4.46726200	1.92092400	-0.29950200
Mg	5.96675600	-0.66341600	-0.22355600
O	0.00210500	-2.51464200	-1.24195500
H	0.00480600	-3.42400500	-0.91689700
H	-0.00237800	-2.53699800	-2.20728000

Final *optimized* geometry of 3_Mg+OH

HF=-1416.82788

H	4.48901400	-1.62482900	1.77566300
H	2.99650700	0.96478900	1.81476800
O	4.42743600	0.17758900	-1.05936900
O	4.56753500	-1.02564800	1.02868700
O	3.03170200	1.38452900	0.92495300
Ni	3.03373700	-0.57562700	-0.02790500

H	1.49988600	-1.62243200	1.78077300
H	0.00763900	0.96716900	1.81954000
O	1.52976500	0.04464500	-1.07923100
O	1.51908300	-1.04541600	1.01246400
O	0.01517500	1.40830600	0.92561800
Ni	0.00017800	-0.70128500	-0.10014900
H	-1.48879100	-1.62005300	1.78588100
H	-2.98119400	0.96942700	1.82492700
O	-1.50621900	0.06204200	-1.06060900
O	-1.49374400	-0.94587400	1.10155800
O	-3.01840900	1.40591100	0.94188800
Ni	-2.98996600	-0.55934200	-0.01021700
H	-4.47786100	-1.61785600	1.79091300
O	-4.36267900	0.19791400	-1.05965100
O	-4.53857900	-1.00993300	1.04431000
Mg	-5.92121200	-0.69292000	-0.31411600
Mg	-4.47869500	1.86289200	-0.24564200
Mg	-1.49011500	1.85958100	-0.24987300
Mg	1.49992200	1.85725100	-0.25513200
Mg	4.48856000	1.85589100	-0.26089900
Mg	5.98040700	-0.73365700	-0.29986500
O	-0.44885200	-2.43868400	-1.13322900
H	0.00011300	-2.83263600	-1.91367000

Final *optimized* geometry of Non-linear 3_Mg

HF=-1417.1490988

H	2.98904800	-0.67594800	1.84984000
O	3.07789600	1.30109900	-0.88449700
O	3.25489200	-0.65266600	0.91509100
H	1.49455200	-3.25882400	1.67859500
H	1.49446200	1.90704000	2.02109200
H	-0.00006700	-0.67587000	1.84988100
O	1.42708100	-1.33680800	-1.14154800
O	-0.00013000	1.28917300	-1.06450700
O	1.56247100	-2.88082000	0.80089900
O	1.53591100	2.32541400	1.13978400
O	-0.00009000	-0.38634500	0.92915500
Ni	1.52379900	0.36994100	-0.17982400
Ni	0.00005100	-2.19403200	-0.09005500
H	-1.49450500	-3.25890100	1.67858300
H	-1.49459300	1.90695800	2.02109200
H	-2.98906700	-0.67610800	1.84981600
O	-1.42709800	-1.33668600	-1.14141400

O	-3.07795000	1.30103700	-0.88461100
O	-1.56221400	-2.88050800	0.80103800
O	-1.53592500	2.32556600	1.13988000
O	-3.25503000	-0.65286600	0.91511700
Ni	-1.52390700	0.36996600	-0.17985200
Mg	-2.98890100	2.90550400	-0.00255700
Mg	0.00003400	2.90523400	-0.00280900
Mg	2.98887800	2.90565500	-0.00254900
Mg	4.48322100	0.32318000	-0.17387900
Mg	-4.48312000	0.32299000	-0.17391300
Mg	-2.98867100	-2.25951700	-0.34510300
Mg	2.98883200	-2.25951000	-0.34515200

Final *optimized* geometry of 3_Mg+H_2O

HF=- 1493.5853064

H	-2.99572600	0.43526600	1.94817400
O	-3.11232700	-1.33033600	-0.91764100
O	-3.27517900	0.50043000	1.01903300
H	-1.44737100	2.99169100	1.96775400
H	-1.55640200	-2.18393100	1.93212200
H	-0.00742100	0.37214600	1.95185800
O	-1.43016400	1.30303200	-1.02597300
O	-0.03578900	-1.37444200	-1.09446200
O	-1.53138900	2.74442000	1.04353600
O	-1.60530500	-2.55625600	1.02950100
O	-0.01115700	0.18373400	1.00219600
Ni	-1.53238000	-0.45521200	-0.16904200
Ni	0.04316500	2.15487500	0.02072700
H	1.54108200	2.92869700	1.97181700
H	1.43184400	-2.24695200	1.93605100
H	2.98036000	0.30920600	1.95614000
O	1.47851700	1.18495800	-1.00973700
O	3.03752700	-1.46609300	-0.91285300
O	1.62763800	2.63770100	1.06564800
O	1.46987400	-2.60956000	1.03008700
O	3.25700000	0.34842600	1.02574100
Ni	1.49560100	-0.54891400	-0.13219400
O	0.36887800	3.86686900	-1.14018500
H	0.37157300	4.73665100	-0.71660300
H	0.17654900	4.00824800	-2.07686500
Mg	2.91097600	-3.12730100	-0.15378400
Mg	-0.07738300	-3.06490000	-0.15641700
Mg	-3.06594900	-3.00135700	-0.16157800

Mg	-4.50578100	-0.38126400	-0.14658300
Mg	4.46005300	-0.57047300	-0.13419700
Mg	3.02031800	2.04960900	-0.11744300
Mg	-2.95709300	2.17609900	-0.12460800

Final *optimized* geometry of Non-linear 3_Mg+OH

HF=-1492.8950685

H	-2.81222200	0.71616900	2.22168000
O	-3.20446400	-1.43974000	-0.64124900
O	-3.30641000	0.80063500	1.37391600
H	-1.32977500	3.28887800	1.87841100
H	-1.31034900	-1.86307200	2.39254200
H	0.17183300	0.70987500	2.04930000
O	-1.61019200	0.98548700	-0.90507200
O	-0.06755800	-1.62153700	-1.02612500
O	-1.43643800	2.80886000	1.06151600
O	-1.41112500	-2.44221200	1.60356800
O	0.23560200	0.50539600	1.10122400
Ni	-1.63761000	-0.79857900	-1.51852800
Ni	0.04031400	2.01953600	0.09714600
H	1.65422100	3.28266600	1.70597500
H	1.67368400	-1.86894200	2.22006200
H	3.15583300	0.70390300	1.87674600
O	1.42832000	1.16564800	-1.05107300
O	3.10700300	-1.36566900	-0.79862300
O	1.60483500	2.95421600	0.79838400
O	1.66289100	-2.28061900	1.33275900
O	3.46238200	0.74567400	0.94755700
Ni	1.42333700	-0.60413000	-0.22605000
O	-0.57489200	3.46410100	-0.80332700
H	-0.16387100	4.32548700	-0.57127700
Mg	3.04902900	-2.93791900	0.14761100
Mg	0.06237700	-2.93014300	0.32070300
Mg	-2.91892400	-2.92530700	0.49281600
Mg	-4.42047200	-0.34649800	0.32180900
Mg	4.53073900	-0.36494900	-0.19544000
Mg	3.02891300	2.21365400	-0.36624800
Mg	-2.93900800	2.22382300	-0.02066500

Final *optimized* geometry of 4_Mg

HF=- 1737.32123299

H	5.97801000	-1.88755000	1.51131500
O	5.90048700	0.26325400	-1.05401900

O	6.05068800	-1.17039000	0.87187500
H	4.48351400	0.67362300	1.88707100
H	2.98906100	-1.88769700	1.51173200
O	2.98914900	0.13333300	-1.08347400
O	4.49977000	1.19834600	1.05626800
O	3.01424700	-1.47773700	0.63625400
Ni	4.52020900	-0.65004200	-0.15770200
H	1.49453700	0.67360000	1.88720100
H	0.00006600	-1.88775200	1.51169500
O	0.00002400	0.17222400	-1.09101400
O	1.50096900	1.16785400	1.03655500
O	0.00002000	-1.49612800	0.63216200
Ni	1.49048300	-0.72013600	-0.22680500
H	-1.49439500	0.67359800	1.88720500
H	-2.98893300	-1.88769200	1.51174200
O	-2.98913800	0.13335300	-1.08347300
O	-1.50101700	1.16786200	1.03655700
O	-3.01420600	-1.47769200	0.63628400
Ni	-1.49045400	-0.72011700	-0.22680200
H	-4.48335500	0.67361400	1.88707700
H	-5.97786200	-1.88755300	1.51132500
O	-5.90049200	0.26324800	-1.05401400
O	-4.49979500	1.19830000	1.05626100
O	-6.05066500	-1.17046700	0.87182100
Ni	-4.52019900	-0.65006200	-0.15770900
Mg	-7.47246000	-0.73145600	-0.42677500
Mg	-5.97787100	1.82985500	-0.05096800
Mg	-2.98944800	1.82984600	-0.05124600
Mg	-0.00003500	1.82943900	-0.05183200
Mg	2.98935500	1.82980400	-0.05124300
Mg	5.97785000	1.82987400	-0.05099300
Mg	7.47243700	-0.73145300	-0.42679200

Final *optimized* geometry of 4_Mg+H_2O

HF=- 1813.73977897

H	6.01550300	-1.70830500	1.70682100
O	5.93777400	0.17379400	-1.06564100
O	6.09065600	-1.06773300	0.99289300
H	4.52830200	0.88165800	1.82592900
H	3.02650700	-1.70059500	1.71857600
O	3.03342800	0.04410400	-1.05985700
O	4.54525100	1.31554200	0.94571600
O	3.04754400	-1.42156100	0.79311100

Ni	4.55396600	-0.64365000	-0.07474400
H	1.53932200	0.88951500	1.83782100
H	0.03771100	-1.69215100	1.73112900
O	0.14689200	0.13755700	-1.09416700
O	1.55484000	1.35635000	0.96630600
O	0.08869000	-0.97533300	1.09742600
Ni	1.54708900	-0.64696000	-0.07136200
H	-1.44963300	0.89740700	1.84975600
H	-2.95147700	-1.68458000	1.74199600
O	-3.06029500	0.12789400	-1.08149300
O	-1.46557100	1.21078000	0.90757200
O	-2.98117400	-1.24837000	0.88288700
Ni	-1.44102300	-0.57602100	-0.15125900
H	-4.43861200	0.90535600	1.86156100
H	-5.94042000	-1.67665900	1.75432400
O	-5.89514600	0.21142100	-1.02061000
O	-4.46160600	1.37712300	0.99909500
O	-6.01381900	-1.03816500	1.03788000
Ni	-4.49003200	-0.57160700	-0.04320600
Mg	-7.44056200	-0.71939800	-0.28562200
Mg	-5.93854000	1.86275900	-0.17821600
Mg	-2.95007100	1.85494200	-0.18991100
Mg	0.03924800	1.84668800	-0.20237600
Mg	3.02818500	1.83923500	-0.21364400
Mg	6.01698500	1.83112500	-0.22573800
Mg	7.50434100	-0.75893400	-0.34492200
O	-1.22919600	-2.10507000	-1.23187900
H	-1.89397600	-2.81290000	-1.19535000
H	-1.05441900	-1.89174200	-2.16534300

Final *optimized* geometry of 4_Mg+OH

HF=-1813.05386007

H	6.01170100	-1.62627100	1.78763500
O	5.94259400	0.12973000	-1.06586900
O	6.08865200	-1.01249000	1.05106000
H	4.54235400	0.97884400	1.78915000
H	3.02372500	-1.60010400	1.80406500
O	3.04929700	0.02405100	-1.05592100
O	4.56027600	1.37989200	0.89667000
O	3.04694400	-1.36067100	0.86771100
Ni	4.55261100	-0.63160600	-0.03956000
H	1.55294000	1.00849800	1.80385600
H	0.03409200	-1.57142100	1.81753200

O	0.15365600	0.10853700	-1.09905900
O	1.57420500	1.39716000	0.90255500
O	0.05333200	-1.02890100	1.02843100
Ni	1.55842300	-0.62256300	-0.05167000
H	-1.43589500	1.03456600	1.81988400
H	-2.95533100	-1.54319100	1.83218200
O	-3.02685000	0.05947100	-1.06325000
O	-1.45967100	1.46006200	0.92059000
O	-3.00254500	-1.26744600	0.90042000
Ni	-1.40210700	-0.82167300	-0.21529600
H	-4.42529900	1.06426200	1.83194100
H	-5.94350800	-1.51297200	1.84479300
O	-5.87079400	0.23646600	-1.00876900
O	-4.45167300	1.43650300	0.92708100
O	-6.02416700	-0.89997200	1.11058500
Ni	-4.51385600	-0.55434000	0.02159100
Mg	-7.43582200	-0.63915200	-0.23282200
Mg	-5.91668600	1.93038800	-0.23578500
Mg	-2.93618900	1.89999800	-0.24958400
Mg	0.05807100	1.87150300	-0.26654300
Mg	3.04814200	1.84307800	-0.27759800
Mg	6.03061000	1.81788700	-0.29373100
Mg	7.49975800	-0.78011200	-0.30420100
O	-1.74046500	-2.49772000	-1.35303300
H	-1.18364400	-3.22757700	-1.70331800

Final *optimized* geometry of Non-linear 4_Mg

HF=- 1813.49157517

H	-4.07260100	-1.49483400	1.73592600
O	-3.74170500	-1.56337300	0.83928000
O	-2.18768800	-1.44818400	-1.12308000
O	0.44815500	-3.18101700	-0.94046900
H	-1.48617300	-2.98888600	1.84877500
O	-1.53552000	-3.32605300	0.93900900
H	-4.07273300	1.49457600	1.73589700
O	-3.74174300	1.56321200	0.83929500
Ni	-3.02203700	-0.00005900	-0.05694800
O	-2.18776600	1.44806700	-1.12306900
Ni	2.10807900	0.00003200	0.03220200
H	3.68556200	0.00011500	2.07455100
O	3.00337500	-1.46924300	-0.75373800
O	4.19745200	0.00009700	1.24429700
Ni	-0.44429000	-1.58139900	-0.18646800

H	1.09973900	-1.49464600	1.96146300
O	0.45253400	0.00007800	-0.96558900
O	1.32259000	-1.56245900	1.04070700
H	-1.48630600	-0.00006800	1.84878400
O	-1.23125600	-0.00004400	0.91621800
O	3.00328400	1.46932100	-0.75378200
Ni	-0.44441700	1.58145000	-0.18637100
H	1.09969500	1.49466000	1.96146400
O	0.44799900	3.18104500	-0.94050000
O	1.32250900	1.56250800	1.04070300
H	-1.48632900	2.98882000	1.84876400
O	-1.53563900	3.32592100	0.93898400
Mg	-3.11975200	2.98888800	-0.30931600
Mg	-0.53388200	4.48352100	-0.19677400
Mg	2.05230500	2.98880200	-0.08389100
Mg	4.63857100	1.49428200	0.02874100
Mg	4.63868500	-1.49408600	0.02873500
Mg	2.05246100	-2.98874700	-0.08385900
Mg	-0.53364600	-4.48357800	-0.19676900
Mg	-3.11964900	-2.98903200	-0.30931500

Final *optimized* geometry of Non-linear 4_Mg+H_2O

HF=- 1889.92047236

H	-3.84506500	-1.49512800	1.94170000
O	-3.58971600	-1.58341900	1.02234200
O	-2.05618900	-1.47999500	-1.02077400
O	0.56760000	-3.18043600	-0.90794700
H	-1.25634900	-2.98933300	1.95313700
O	-1.34405000	-3.33505800	1.04987500
H	-3.84552000	1.49381400	1.94173200
O	-3.58998700	1.58230300	1.02247700
Ni	-2.97210300	-0.00040800	0.00512700
O	-2.05639600	1.47943100	-1.02069000
Ni	2.25732500	0.00023700	-0.00876900
H	3.92044900	0.00050600	1.97625700
O	3.12897100	-1.46979200	-0.82406800
O	4.40884200	0.00055900	1.13087200
Ni	-0.30842300	-1.57359300	-0.11409300
H	1.33212600	-1.49449900	1.96474000
O	0.56465800	0.00009400	-0.93324500
O	1.51818500	-1.56231500	1.03642800
H	-1.25677800	-0.00030300	1.95319200
O	-1.08660100	-0.00024700	0.99766000

O	3.12850600	1.47052100	-0.82408300
Ni	-0.30882900	1.57348300	-0.11392700
H	1.33171800	1.49469900	1.96477400
O	0.56670600	3.18055200	-0.90790800
O	1.51775400	1.56260000	1.03646600
H	-1.25722600	2.98881700	1.95320000
O	-1.34497500	3.33447300	1.04992700
O	-4.68318300	0.00101600	-1.21280300
H	-5.56863500	0.00058100	-0.82784500
H	-4.77705000	-0.00008800	-2.17297200
Mg	-2.97305700	2.98831400	-0.13952500
Mg	-0.38545700	4.48326700	-0.12767000
Mg	2.20310100	2.98867100	-0.11622600
Mg	4.79187000	1.49455300	-0.10468200
Mg	4.79236500	-1.49330000	-0.10472800
Mg	2.20398400	-2.98817100	-0.11627700
Mg	-0.38412100	-4.48351900	-0.12776400
Mg	-2.97216700	-2.98927600	-0.13959500

Final *optimized* geometry of Non-linear 4_Mg+OH

HF=-1889.2122275

H	-3.84506500	-1.49512800	1.94170000
O	-3.58971600	-1.58341900	1.02234200
O	-2.05618900	-1.47999500	-1.02077400
O	0.56760000	-3.18043600	-0.90794700
H	-1.25634900	-2.98933300	1.95313700
O	-1.34405000	-3.33505800	1.04987500
H	-3.84552000	1.49381400	1.94173200
O	-3.58998700	1.58230300	1.02247700
Ni	-2.97210300	-0.00040800	0.00512700
O	-2.05639600	1.47943100	-1.02069000
Ni	2.25732500	0.00023700	-0.00876900
H	3.92044900	0.00050600	1.97625700
O	3.12897100	-1.46979200	-0.82406800
O	4.40884200	0.00055900	1.13087200
Ni	-0.30842300	-1.57359300	-0.11409300
H	1.33212600	-1.49449900	1.96474000
O	0.56465800	0.00009400	-0.93324500
O	1.51818500	-1.56231500	1.03642800
H	-1.25677800	-0.00030300	1.95319200
O	-1.08660100	-0.00024700	0.99766000
O	3.12850600	1.47052100	-0.82408300
Ni	-0.30882900	1.57348300	-0.11392700

H	1.33171800	1.49469900	1.96477400
O	0.56670600	3.18055200	-0.90790800
O	1.51775400	1.56260000	1.03646600
H	-1.25722600	2.98881700	1.95320000
O	-1.34497500	3.33447300	1.04992700
O	-4.68318300	0.00101600	-1.21280300
H	-5.56863500	0.00058100	-0.82784500
H	-4.77705000	-0.00008800	-2.17297200
Mg	-2.97305700	2.98831400	-0.13952500
Mg	-0.38545700	4.48326700	-0.12767000
Mg	2.20310100	2.98867100	-0.11622600
Mg	4.79187000	1.49455300	-0.10468200
Mg	4.79236500	-1.49330000	-0.10472800
Mg	2.20398400	-2.98817100	-0.11627700
Mg	-0.38412100	-4.48351900	-0.12776400
Mg	-2.97216700	-2.98927600	-0.13959500

Final *optimized* geometry of 5_Mg
HF=- 2133.51733112

H	7.47260300	-1.90737900	1.49218000
O	7.38870100	0.24119200	-1.07001700
O	7.54688300	-1.18948700	0.85392400
H	5.97808900	0.65341300	1.87034500
H	4.48359600	-1.90710500	1.49211900
O	4.49676100	0.10508200	-1.10896200
O	5.99351700	1.16612200	1.03250800
O	4.51289800	-1.51950500	0.60424800
Ni	6.02417700	-0.67836300	-0.17209500
H	2.98904300	0.65322700	1.87046900
H	1.49459100	-1.90760000	1.49242100
O	1.46201200	0.15886800	-1.11705600
O	3.00995500	1.15403800	1.01714500
O	1.48931200	-1.20113600	0.84612100
Ni	2.97871000	-0.72154600	-0.23308400
H	0.00004700	0.65314100	1.87055500
H	-1.49444300	-1.90763500	1.49241900
O	-1.46198600	0.15886200	-1.11711800
O	-0.00005500	1.20930300	1.04961200
O	-1.48923600	-1.20146800	0.84579700
Ni	0.00005400	-0.65049100	-0.19483500
H	-2.98895000	0.65316000	1.87047300
H	-4.48345500	-1.90720600	1.49212500

O	-4.49658900	0.10516700	-1.10913800
O	-3.00998000	1.15394200	1.01712500
O	-4.51274300	-1.51821000	0.60493500
Ni	-2.97862800	-0.72129300	-0.23299300
H	-5.97797500	0.65326400	1.87034800
H	-7.47243100	-1.90755300	1.49218900
O	-7.38883800	0.24103700	-1.07009900
O	-5.99362900	1.16604700	1.03253500
O	-7.54685200	-1.18975000	0.85383300
Ni	-6.02418900	-0.67839100	-0.17217400
Mg	-8.96686400	-0.74909800	-0.44513800
Mg	-7.47217800	1.81132900	-0.06644800
Mg	-4.48396300	1.81144200	-0.06610900
Mg	-1.49493400	1.81190100	-0.06744800
Mg	1.49479100	1.81191900	-0.06745200
Mg	4.48381100	1.81152300	-0.06612100
Mg	7.47208100	1.81151500	-0.06644200
Mg	8.96682000	-0.74888400	-0.44515000

Final *optimized* geometry of 5_Mg+H_2O

HF=- 2209.9411317

H	7.47204300	-1.64458600	1.74948600
O	7.39031700	0.18670600	-1.05212900
O	7.54462600	-1.01253700	1.02611400
H	5.97738300	0.94289800	1.82049400
H	4.48303700	-1.64495800	1.74934900
O	4.48805600	0.05234800	-1.06767100
O	5.99471100	1.35750200	0.93057600
O	4.50505700	-1.37810700	0.81907400
Ni	6.01278900	-0.61561000	-0.05320600
H	2.98844900	0.94266900	1.82045200
H	1.49408500	-1.64433400	1.74960000
O	1.52597900	0.10029400	-1.11299700
O	3.01344500	1.33997200	0.91692700
O	1.49440900	-1.02002900	1.01937900
Ni	2.99421600	-0.64752100	-0.08527300
H	-0.00055800	0.94261600	1.82040800
H	-1.49492800	-1.64462900	1.74943200
O	-1.52666700	0.09904900	-1.11347900
O	-0.00102400	1.42497900	0.94586500
O	-1.49515000	-1.02623500	1.01415500
Ni	0.00067100	-0.73413000	-0.17834900
H	-2.98954200	0.94242600	1.82037000

H	-4.48392400	-1.64531300	1.74923000
O	-4.48906900	0.05230400	-1.06780700
O	-3.01452700	1.33871900	0.91649700
O	-4.50615700	-1.37839200	0.81900500
Ni	-2.99590500	-0.64809100	-0.08573800
H	-5.97847000	0.94237100	1.82031100
H	-7.47288900	-1.64525200	1.74925600
O	-7.39136300	0.18602700	-1.05237500
O	-5.99594100	1.35706900	0.93044000
O	-7.54561700	-1.01323500	1.02582900
Ni	-6.01377800	-0.61603400	-0.05346400
Mg	-8.96753600	-0.72539100	-0.31194300
Mg	-7.47305600	1.86221600	-0.24064900
Mg	-4.48449600	1.86244700	-0.24053300
Mg	-1.49497700	1.86222800	-0.24067300
Mg	1.49365800	1.86234300	-0.24061000
Mg	4.48315100	1.86280100	-0.24036000
Mg	7.47178000	1.86288600	-0.24041600
Mg	8.96648900	-0.72458600	-0.31163300
O	0.02128900	-2.46481200	-1.25196100
H	0.00167000	-3.38968900	-0.97259900
H	0.02069900	-2.44774800	-2.21864600

Final *optimized* geometry of 5_Mg+OH

HF=-2209.24943894

H	7.48140700	-1.69165100	1.71383200
O	7.39916700	0.18144300	-1.05892100
O	7.55406100	-1.04681100	1.00138100
H	5.98952000	0.89597400	1.82517600
H	4.49242800	-1.68866800	1.71511700
O	4.49735000	0.05206300	-1.07583700
O	6.00616600	1.32387200	0.94141900
O	4.51638100	-1.40540500	0.78995300
Ni	6.02390300	-0.63419100	-0.07183000
H	3.00051400	0.89899500	1.82640700
H	1.50331100	-1.68576200	1.71562300
O	1.52046500	0.09150700	-1.11344000
O	3.02392700	1.30303300	0.92624100
O	1.50045800	-1.08027600	0.96971800
Ni	3.00609700	-0.66427100	-0.10412200
H	0.01150300	0.90196400	1.82760000
H	-1.48553900	-1.68277000	1.71804100
O	-1.49367100	0.11203200	-1.10508600

O	0.01823300	1.39699800	0.96141300
O	-1.48018900	-0.99809400	1.04459800
Ni	0.00044100	-0.69725100	-0.16121500
H	-2.97747200	0.90492100	1.82871900
H	-4.47458800	-1.67999000	1.71857500
O	-4.47991700	0.05296700	-1.07337900
O	-3.00416300	1.31964400	0.93114700
O	-4.49750700	-1.39380900	0.79369700
Ni	-2.97178900	-0.65967300	-0.09874600
H	-5.96640300	0.90786100	1.82991200
H	-7.46354500	-1.67678600	1.71974300
O	-7.38033100	0.19625200	-1.05269200
O	-5.98347200	1.33296200	0.94466800
O	-7.53658300	-1.03308600	1.00708600
Ni	-6.00778300	-0.62535700	-0.06512900
Mg	-8.95807200	-0.72393600	-0.32639200
Mg	-7.46090700	1.86076900	-0.21616000
Mg	-4.47208800	1.85790800	-0.21755400
Mg	-1.48275800	1.85496700	-0.21878900
Mg	1.50617200	1.85177800	-0.21972600
Mg	4.49503100	1.84902100	-0.22064400
Mg	7.48402100	1.84586100	-0.22216600
Mg	8.97595300	-0.74178000	-0.33353500
O	-0.49735900	-2.35966400	-1.28776100
H	-0.04785000	-2.76338700	-2.06363700

Final *optimized* geometry of Non-linear 5_Mg

HF=- 2057.69761025

H	4.48279800	-2.75625900	1.65122000
O	4.48383800	-0.81889500	-1.06771700
O	4.63626000	-2.15808500	0.93115500
H	2.99114800	-0.17429200	1.85465200
H	1.49392400	-2.75199200	1.64790700
H	1.50073900	2.40802800	2.05806500
O	3.14131400	1.91511200	-0.88783500
O	1.58919400	-0.90362500	-1.05125700
O	3.07532100	0.13086800	0.96433100
O	1.57419600	-2.45460200	0.73557700
O	1.53042500	2.89162000	1.20471800
Ni	3.09538500	-1.63363700	-0.07559000
Ni	1.56413700	0.92475700	-0.19022100
H	0.00319300	-0.16995300	1.85144100
H	-1.49496000	-2.74753800	1.64483500

H	-1.48715300	2.41251000	2.05499200
O	-0.10401200	1.80167100	-1.00260500
O	-1.60282500	-0.91179500	-1.04116300
O	-0.01413200	0.06588600	0.90762700
O	-1.58623900	-2.46480200	0.72627000
O	-1.51807300	2.89116200	1.20196300
Ni	0.00007600	-1.72661600	-0.15514000
Ni	-1.56299800	0.89588000	-0.13374200
H	-2.98491800	-0.16540700	1.84847000
H	-4.48385200	-2.74285200	1.64198800
O	-3.07823900	1.89310600	-0.86328900
O	-4.47721500	-0.80110500	-1.07058800
O	-3.06695500	0.13650400	0.95809500
O	-4.63566900	-2.13922200	0.92779500
Ni	-3.10373500	-1.63472600	-0.08159200
Mg	5.98201000	-1.73336100	-0.35881800
Mg	-5.97607200	-1.71550400	-0.37102400
Mg	4.49173800	0.84987700	-0.15672200
Mg	-4.47839300	0.86338800	-0.16617800
Mg	0.01028100	3.44093100	0.04530500
Mg	3.00114000	3.43361700	0.04754800
Mg	-2.98059200	3.44262900	0.04137400

Final *optimized* geometry of Non-linear 5_Mg+H_2O

HF=- 2134.126886714

H	4.48355400	-2.54638100	1.83951400
O	4.48527100	-0.75385800	-0.97082300
O	4.63647300	-1.98304800	1.09521000
H	2.98893900	0.04127800	1.90230800
H	1.49453600	-2.54638600	1.83940000
H	1.49454500	2.62900900	1.96520900
O	3.07652400	1.94894400	-0.92883200
O	1.64311400	-0.85449900	-0.95333300
O	3.06399300	0.30636000	1.00030100
O	1.61103500	-2.34790400	0.90003100
O	1.53128100	3.08743500	1.10023200
Ni	3.11007300	-1.51777400	0.06440700
Ni	1.53628600	0.99821600	-0.15091100
H	0.00008600	0.04164500	1.90232900
H	-1.49438000	-2.54637500	1.83940100
H	-1.49434900	2.62902000	1.96520800
O	-0.00026700	1.84968700	-1.03730700
O	-1.64311800	-0.85455700	-0.95330400

O	-0.00004500	0.17953900	0.93907500
O	-1.61101500	-2.34792600	0.90002800
O	-1.53127900	3.08735800	1.10019600
Ni	0.00002300	-1.76368500	-0.13203300
Ni	-1.53629000	0.99814100	-0.15088100
H	-2.98876500	0.04129700	1.90230600
H	-4.48340200	-2.54634900	1.83951300
O	-3.07637400	1.94884900	-0.92874000
O	-4.48529700	-0.75381600	-0.97079000
O	-3.06396600	0.30635700	1.00028400
O	-4.63646100	-1.98316300	1.09513600
Ni	-3.11009000	-1.51780700	0.06441300
Mg	5.97818500	-1.63313800	-0.22471700
Mg	-5.97823000	-1.63309300	-0.22472200
Mg	4.48370300	0.95479000	-0.16196000
Mg	-4.48373100	0.95482500	-0.16196000
Mg	-0.00000100	3.54242500	-0.09921900
Mg	2.98923200	3.54306100	-0.09911600
Mg	-2.98924100	3.54308700	-0.09912100
O	0.00015700	-3.37667000	-1.40097700
H	0.00016400	-4.30258900	-1.13118800
H	-0.00016400	-3.34887900	-2.36518800

Final *optimized* geometry of Non-linear 5_Mg+OH

HF=-2133.420002046

H	4.48418200	-2.55251400	1.80885100
O	4.47883700	-0.73378200	-0.98581800
O	4.63664900	-1.98343000	1.06878200
H	2.97873800	0.02826800	1.89503600
H	1.49528400	-2.56524800	1.80933400
H	1.47341800	2.60918100	1.98145500
O	3.05310500	1.95269500	-0.89913100
O	1.63438200	-0.84069100	-0.95593900
O	3.05755600	0.29440400	0.99469200
O	1.61286300	-2.35133700	0.87479800
O	1.50377200	3.05954400	1.11401100
Ni	3.11069700	-1.52220200	0.03867900
Ni	1.54173700	0.98597400	-0.11949600
H	-0.00998500	0.01594600	1.89584500
H	-1.49371600	-2.57797400	1.81018700
H	-1.51560100	2.59652400	1.98222600
O	0.09750800	1.84236900	-1.03169700
O	-1.63192400	-0.87863600	-0.97819000

O	0.00618900	0.16705100	0.93227200
O	-1.60318900	-2.37166300	0.87246300
O	-1.54502800	3.05119200	1.11375500
Ni	-0.00082600	-1.74455800	-0.12626200
Ni	-1.56319500	0.98792700	-0.19680800
H	-2.99903800	0.00297200	1.89665400
H	-4.48252200	-2.59047100	1.81124900
O	-3.14173900	1.95841400	-0.93240200
O	-4.48830300	-0.77889700	-0.98623300
O	-3.08310500	0.26552100	0.99501100
O	-4.63970200	-2.03584900	1.06158500
Ni	-3.11116900	-1.54127800	0.04455200
Mg	5.97455100	-1.61450400	-0.24803700
Mg	-5.98211100	-1.66512800	-0.24500100
Mg	4.46846300	0.96655000	-0.16116400
Mg	-4.49801100	0.92842900	-0.15851800
Mg	-0.02562800	3.53332400	-0.07417100
Mg	2.96335200	3.54768500	-0.07423600
Mg	-3.01459100	3.52231300	-0.07259600
O	0.33741700	-3.37561800	-1.36562200
H	-0.20821200	-4.16514500	-1.56350300

SI-4. Example of input file

%chk=**3_Mg**

%mem=2GB

%nproc=6

#P PBE1PBE/gen opt=(modred,maxcycle=1000) freq pseudo=read scf=xqc

optimization[RD1]

4 2

O	-3.45146000	2.98903000	5.62170000
H	-4.31431000	1.49453000	8.67981000
H	-4.31431000	4.48353000	8.67981000
O	-4.31431000	1.49453000	7.58460000
O	-4.31431000	4.48353000	7.58460000
Ni	-5.17716000	2.98903000	6.59443000
O	-6.04001000	1.49453000	5.62170000
H	-6.90286000	2.98903000	8.67981000
O	-6.90286000	2.98903000	7.58460000
Mg	-2.58861000	4.48353000	6.59443000
Mg	-2.58861000	1.49453000	6.59443000

Mg	-5.17716000	0.00003300	6.59443000
Mg	-7.76571000	1.49453000	6.59443000

10 F
11 F
12 F
13 F
2 F
3 F
8 F

Ni
LANL2DZ
****
O H
6-311++G**
****

Ni Mg
LANL2DZ

SI-4. References

1. Rosso, K.M., Smith, D.M.A., Dupuis, M. An ab initio model of electron transport in hematati α-Fe_2O_3 basal planes. *J. Chem. Phys.* **2003**, *118*, 6455–6466.
2. Wadt, W.R.; Hay, P.J. Ab initio effective core potentials for molecular calculations, Potentials for main group elements Na to Bi. J. Chem. Phys. **1985**, *82*, 284–298.
3. Dennington, R., Keith, T., Millam, J. GaussView, Version 5, Semichem Inc. Shawnee Mission, KS, 2009.
4. Li YF, Selloni A. Mechanism and Activity of Water Oxidation on Selected Surfaces of Pure and Fe-Doped NiOx. ACS Catal. **2014**, *4*, 1148–1153.
5. Biesinger, M.C.; Payne, B.P.; Lau, L.W.M.; Gerson, A.; Smart, R.S.C. X-ray Photoelectron Spectroscopic Chemical State Quantification of Mixed Nickel Metal, Oxide and Hydroxide Systems. *Surf. Interface Anal.* **2009**, *41*, 324–332.
6. Grosvenor, A.P.; Biesinger, M.C.; Smart, R.S.C.; McIntyre, N.S. New Interpretations of XPS Spectra of Nickel Metal and Oxides. *Surf. Sci.* **2006**, *600*, 1771−1779.
7. Dennington, R.; Keith, T.; Millam, J. Semichem Inc., Shawnee Mission, KS, 2009.
8. Li, Y.F.; Selloni, A. Mosaic Texture and Double c-Axis Periodicity of β-NiOOH: Insights from First-Principles and Genetic Algorithm Calculations. *J. Phys. Chem. Lett.* **2014**, *5*, 3981–3985.

© 2017 by the authors. Submitted for possible open access publication under the terms and conditions of the Creative Commons Attribution (CC-BY) license (http://creativecommons.org/licenses/by/4.0/).
